# Wiener index application in intuitionistic fuzzy rough graphs for transport network flow

**DOI:** 10.1038/s41598-025-94488-y

**Published:** 2025-03-20

**Authors:** Noorjahan Shaik, Sharief Basha Shaik

**Affiliations:** Department of Mathematics, School of Advanced Sciences, Vellore Institute of Technology, Vellore, Tamil Nadu 632014 India

**Keywords:** Intuitionistic fuzzy rough graph (IFRG), Wiener index, Connectivity index, Transport network flow, Chemistry, Mathematics and computing

## Abstract

The applicability of different topological indices is indispensable in fields such as chemistry, electronics, economics, business studies, medicine, and the social sciences. The most popular index in graph theory is the wiener index $\left(\mathcal{W}\mathcal{I}\right)$, which is based on the geodesic distance between two vertices. It is assumed that the weight of the geodesic between vertex *x* and vertex *y* in intuitionistic fuzzy rough graphs (IFRG) is zero in the absence of a directed path. With regard to intuitionistic fuzzy rough graphs, the objective of this work is to investigate in detail the wiener index $\left(\mathcal{W}\mathcal{I}\right)$ and the average wiener index ($\mathcal{A}\mathcal{W}\mathcal{I})$. Also, the connectivity index $\left(\mathcal{C}\mathcal{I}\right)$ is one of the most significant indices, providing several examples and results. For intuitionistic fuzzy rough graphs, alternative distance and degree-based topological indices have also been developed. The research on intuitionistic fuzzy rough graphs that has been suggested is appropriate for representing imprecise data and uncertainty in practical situations. Additionally, examined is the connection between the wiener and connectivity indices. Finally, we proposed the use of wiener indices in transport network flow.

## Introduction

Regarding many real-world issues, we only have partial information. The first person to present a publication on fuzzy sets (FSs) was Zadeh^1^ in *1965*. By assigning membership grades to each item in the set inside the interval [*0, 1*], he expanded the concept of the crisp set. He developed several notions of ordinary sets specific to FS. The intuitionistic fuzzy set (IFSs), a novel set created by Atanassov^2^, was the first to generalise FS by taking non-membership information into account. Researchers began to generalise different FS principles into the context of IFS after taking this into account. Chen et al.^3^ introduce a unique Video Processing (VIPROC) algorithm that uses temporal intuitionistic fuzzy sets (TIFSs) to enhance videos, potentially useful in engineering applications like motion tracking and traffic detection systems.

Pawlak^4^ first put out the notion of rough sets (RSs) as a formal instrument for representing and handling missing data. Knowledge concealed in information systems may be uncovered and articulated as decision rules by applying the ideas of lower and upper approximations from rough set theory. In binary relations^5^ between universal sets *U* and *V*, the research investigates solitary sets, rough set qualities, Boolean equation solutions, Dempster-Shafer theory, fuzzy environments, and many criteria for decision-making. Dubois and Parde^6^ argue that FS and RS serve different purposes and suggest combining them to better understand uncertainty. It suggests deriving upper and lower approximations of fuzzy sets, transforming equivalence relations into fuzzy similarity relations, and unifying independent works. Chakrabarty et al.^7^ develop a measure of fuzziness in rough sets (FRSs) and provide some characterizations and instances of this measure.

The dynamic graph transformer-based parallel framework, a novel approach to detecting system anomalies in cloud infrastructures, utilizes transformers and graph neural networks for improved accuracy and timeliness^8^. Rosenfeld^9^ provides fuzzy analogues of graph-theoretic notions like bridges and trees and investigates the features of fuzzy relations, extending to FS. Borzooei and Rashmanlou^10^ introduce Cayley interval-valued fuzzy graphs, defining their graph theoretic and algebraic properties, including α-connectedness, weakly connectedness, semi-connectedness, locally connectedness, quasi-connectedness, and strength of connectivity. Ashraf et al.^11^ explores regular graphs in a single valued neutrosophic environment, proposing new concepts, properties, and applications in multi-attribute decision making. New operations on vague graphs, such as rejection, maximal product, symmetric difference, and residue product, as well as their features, such as vertex degree and applications in medical diagnostics, are explored by Shao et al.^12^. Complex pythagorean fuzzy sets are introduced in graph theory by Shoaib et al.^13^, who also define features such as composition, cartesian product, strong product, semi-strong product, and direct product. Parvathi and Karunambigai^14^ extended the concept of an FG to encompass an IFG. Shao et al.^15^ discuss the use of bondage set, non-bondage set, bondage number, and non-bondage number concepts in intuitionistic fuzzy graphs, enhancing daily life efficiency and productivity. Qiang et al.^16^ introduce concepts like covering, matching, and dominance in interval-valued intuitionistic fuzzy graphs (IVIFGs), calculate strong node covering and independent numbers, and present their application in social networks. Rao et al.^17^ introduce new intuitionistic fuzzy models in neural networks, computer networks, and clustering, highlighting their precision, flexibility, and compatibility. Mathew et al.^18^ introduce vertex rough graphs, discuss basic graph theoretic definitions, use Pawlak’s rough set theory to create a matrix, and extend rough precision and similarity degree concepts. Akram et al.^19^ fuzzy rough digraphs (FRDGs) and methods for decision-making issues including city selection and mobile phone jammer position recognition by extending fuzzy rough set theory to graphs. Akram and Zafar^20,21^ discuss the use of rough fuzzy digraphs (RFDs) as a mathematical tool for handling vague information, discussing complements, isomorphisms, irregularities, and hybrid decision-making methods. Akram et al.^22^ explore fuzzy rough set theory in graph theory, introduce FRDG as a new structure, discuss their theoretical properties, and discuss their application in decision-making.

Variations in the axiom sets of lower and upper intuitionistic fuzzy set-theoretic operators ensure that distinct kinds of intuitionistic fuzzy relations exist and yield identical operators. Intuitionistic fuzzy rough sets (IFRS) were described by Cornelis et al.^23^. The pair of upper and lower intuitionistic fuzzy rough approximation operators derived from an arbitrary intuitionistic fuzzy relation were examined by Zhou and Wu^24^. In the publications^25^, Wu also worked on IFRS. Zhang et al.^26^ investigate IFRS, providing a new definition, analysis of properties, approximation operators, and connections with special intuitionistic fuzzy relations. Haq et al.^27^ tackle semantic issues with incomplete information by classifying types, introducing a complete system, proposing a fuzzy decision table, and developing a rule extraction method. Mazarbhuiya and Shenify^28^ propose a hybrid approach that combines RS and IFS theory for anomaly detection in computer networks and databases, achieving high true positive rates. Mahmood et al.^29^ introduce innovative techniques for precise disease diagnosis using IFRS, including confidence-level operators, an algorithm, and a medical diagnosis model, demonstrating their effectiveness through comparative analysis. This article^30^ introduces IFRS based aggregation operators (AOs) for multi-attribute group decision-making (MAGDM) problems, examines their properties, and compares them with existing AOs.

Malik and Akram^31^ present techniques for building and improving an effective algorithm for decision-making issues, investigating the application of intuitionistic fuzzy rough models to graphs. In^32^ order to provide flexible and expressive modelling for information systems, the research work presents IFRG, which combine fuzzy and rough sets. It also presents applications to decision-making issues and develops effective algorithms. The intuitionistic fuzzy graph of n^th^ type (IFGNT), a generalisation of IFG and second type IFG, is introduced in this article along with its benefits and uses in social networks^33^. This monograph^34^ investigates hybrid models, especially fuzzy digraphs, for tackling complicated problems and highlights how they might be used in decision-making to overcome uncertainty problems with standard approaches. Zhu^35^ explores the application of deep reinforcement and autonomous learning techniques in an adaptive agent decision model to improve decision-making in complex environments. Yin et al.^36^ introduce the Convolution-based Efficient Transformer Image Feature Extraction Network (CEFormer), an improved model that enhances image feature extraction accuracy and computational speed. Mahmood et al.^37^ introduce an EDAS technique for robotics data handling, utilizing intuitionistic fuzzy rough numbers (IFRN) and new aggregation operators, and showcase its effectiveness through an algorithm and comparative analysis. Tiwari et al.^38^, in order to improve the prediction of phospholipidosis positive molecules, present a unique intuitionistic fuzzy (IF) aided mutual information concept. This concept efficiently handles noise, uncertainty, and ambiguity in real-valued datasets. Sombor indices are used to study intuitionistic fuzzy graphs, which are generalisations of fuzzy graphs. Intuitionistic fuzzy-based topological indices are used to evaluate vaccination centres during the epidemic, emphasising their usefulness and efficiency^39^. Noorjahan and Sharief basha^40^ employs intuitionistic fuzzy rough models to handle complex uncertainty in graph-based models, integrates attribute decision-making, calculates laplacian energy, and proposes a new approach for ranking alternatives. Guo and Das^41^ introduces generalized Reynolds operators on Leibniz algebras, defines their cohomology, discusses formal deformations, and explores the underlying structure of NS-Leibniz algebras.

The association between a chemical structure and several physical qualities, biological activity, and chemical reactivity is determined by topological indices (TIs), which are scalars connected to a molecular graph and properties. The wiener index is the first TI proposed by Wiener in *1947*. This TI is distance-based. Several degree-based TIs have been shown to be able to identify different drug and chemical features. These include the Randic index, Harmonic index, Zagreb indices, Geometric Arithmetic index, Enhanced Zagreb index, and Atomic Bond Connectivity index. The wiener index^42^ of a graph *G* is determined by the sum of vertices distances and can be calculated using various algorithms, including linear time and arbitrary graphs. According to Mehdi Eliasia^43^ the wiener index of graphs is determined through operations like Mycielski’s construction, generalised hierarchical product, and t^th^ subdivision of graphs. The connectivity index ($\mathcal{C}\mathcal{I}$) and wiener index ($\mathcal{W}\mathcal{I}$) for FG with applications were presented by Binu et al.^44^. Islam and Pal^45^ introduce the fuzzy hyper-wiener index (FHWI), a topological index utilized in molecular chemistry, network theory, and physical worlds, and its application in fuzzy graphs and share markets. Islam and Pal studied first zagreb index^46^ and hyper-connectivity index^47^ for fuzzy graph. Islam and Pal^48^ zagreb index (ZI) are crucial tools in network theory, spectral graph theory, molecular chemistry, and mathematics, especially in octane isomers, for accurate acentric factor and entropy estimation. Javaid et al.^49^ explores the second zagreb coindex of F-sum graphs, utilizing zagreb indices and coindices of factor graphs, subdivision-related operations, and the Cartesian product of graphs. Islam and Pal^50^ explore the zagreb index for fuzzy graphs, its applications in real-life problems, and its relevance in india’s kidnapping and abduction cases. Dinar et al.^51^ explore the use of wiener index, distance, degree, and average wiener index in water supply management. This^52^ research explores the concept of the Wiener index (WI) using intuitionistic fuzzy graphs, investigates its bounds, and proposes its application in transport network flow. Ahmad and Nawaz^53^ introduce the wiener index and average Wiener index of directed rough fuzzy graphs (DRFGs), explore their connection to the connectivity index, introduce the complete DRFG concept, and discuss vertices types. Akram and Zafar^54^ determine which nations are most useful for human trafficking. This research study examines connections in fuzzy graphs and rough fuzzy digraphs (RFDs), examines arc features, and applies measurements to actual networks. The edge F-index, a correlational index for fuzzy graphs, is utilized in mathematical chemistry and is strongly linked to the properties of octane isomers and alkanes^55^. Fuzzy graphs^56^, particularly vague graphs (VGs), are crucial in fields like computer science, psychology, medicine, and political sciences for identifying effective individuals and suitable construction sites. Shao et al.^57^ introduce the concept of a perfectly regular vague graph, discuss strongly edge irregular and totally irregular vague graphs, compare them, and apply them to travel time. Yin et al.^58^ introduce an adaptive feature fusion block (AFB) for feature extraction, enhancing generalization and expression abilities by combining dynamic convolution, attention mechanisms, and pixel-based gating mechanisms. Sokolov and Barakhtenko^59^ present a methodology for optimizing pipeline network transmission capacity in tree-shaped water pipelines, using a versatile network model, branch modeling, and a dynamic programming algorithm. Nyende-Byakika^60^ states that in order to distribute water at the necessary pressure and discharge, an optimized distribution system is essential, as demonstrated by recent research that emphasises the significance of water distribution and supply networks in water supply management. Noorjahan and Shaik S. B^61^ introduces a fuzzy rough framework, a hybrid approach combining fuzzy sets and rough sets, for cooperative decision-making in cotton seedlings, enhancing fuzzy understanding.

### Motivation and contribution of this study

Real-world networking in the presence of insufficient data or a rough universe is too complex for fuzzy rough graphs to manage. Thus, this limitation provides an avenue for the introduction of intuitionistic fuzzy rough graphs. The intuitionistic fuzzy rough model is a novel and singular hybrid model intended to manage more complex scenarios, including uncertainty. By characterizing the features of graphs, topological indices based on graph connectivity analysis help in decision-making. When a study of the connectivity index is not adequate to determine the characteristics of graphs with the same connectivity index values, the wiener index may be used in place of the connectivity index in the IFRG. Moreover, although both fuzzy and crisp graph theory recognize the concept of the wiener index, not all graphical structures, including IFRGs, can be captured by these models as they currently stand. Therefore, the purpose of this study is to extend the concept of $\mathcal{W}\mathcal{I}$ to IFRGs. We are motivated by the lack of documentation in the literature about the wiener index ($\mathcal{W}\mathcal{I}$) and average wiener index ($\mathcal{A}\mathcal{W}\mathcal{I}$) of IFRGs. These indices will enable a thorough analysis of various IFRG components. We suggest these ideas for IFRGs as a result. Additionally, we examine the relationship between the wiener index ($\mathcal{W}\mathcal{I})$ and connectivity index ($\mathcal{C}\mathcal{I}$) of IFRGs by providing various instances and findings. The aim of this effort is to tackle hard problems that intuitionistic fuzzy rough graphs have trouble solving.

### Novelty of this study

The novelty of this research lies in the exploration and detailed analysis of the wiener index and the average wiener index, specifically within the framework of intuitionistic fuzzy rough graphs. Additionally, the study introduces alternative distance and degree-based topological indices for IFRGs, which have not been previously developed or explored. The application of these indices in the field of transport network flow represents a novel practical application of the theoretical findings.

### Structure of this study

The following describes the setting of this study article: For the purpose of developing the content, we offer fundamental IFRG definitions, results, and expressions in Section "Preliminaries". We address $\mathcal{W}\mathcal{I}$ of IFRGs and associated findings in Section "Wiener index of an intuitionistic fuzzy rough graph". In Section "Relationship between connectivity index and wiener index of an intuitionistic fuzzy rough graph", we provide the average wiener index and discuss the relation between $\mathcal{C}\mathcal{I}$ and $\mathcal{W}\mathcal{I}$ of an IFRG. We describe an intuitionistic fuzzy rough graph with an example in Section "Some other indices of an intuitionistic fuzzy rough graph", along with a few more indices. The wiener index is applied; this is covered with application in Section "Removal of vertices from an intuitionistic fuzzy rough graph and its impact on the wiener index and average wiener index". Section "Conclusion" concludes this research.

## Preliminaries

This section contains definitions and basic concepts related to intuitionistic fuzzy rough graphs. Perhaps the majority of them can be located in^52,53^.

### Definition 2.1^31^

A pair $\mathcal{G}=\left(\underline{\mathcal{G}},\overline{\mathcal{G}}\right)=\left(RY,SZ\right)$ is an intuitionistic fuzzy rough graph on nonempty set $P$. For $\mathcal{G}=\left(\underline{\mathcal{G}},\overline{\mathcal{G}}\right),$ where $\underline{\mathcal{G}}=\left(\underline{R}Y,\underline{S}Z\right)$ is a lower approximation and $\overline{\mathcal{G}}=\left(\overline{R}Y,\overline{S}Z\right)$ is an upper approximation of the intuitionistic fuzzy rough graph as follows:

$${\left(\underline{S}Z\right)}^{M}\left({p}_{1}{p}_{2}\right)\le min\left\{{\left(\underline{R}Y\right)}^{M}\left({p}_{1}\right),{\left(\underline{R}Y\right)}^{M}\left({p}_{2}\right)\right\}, {\left(\underline{S}Z\right)}^{N}\left({p}_{1}{p}_{2}\right)\le max\left\{{\left(\underline{R}Y\right)}^{N}\left({p}_{1}\right),{\left(\underline{R}Y\right)}^{N}\left({p}_{2}\right)\right\},$$

$${\left(\overline{S}Z\right)}^{M}\left({p}_{1}{p}_{2}\right)\le min\left\{{\left(\overline{R}Y\right)}^{M}\left({p}_{1}\right),{\left(\overline{R}Y\right)}^{M}\left({p}_{2}\right)\right\}, {\left(\overline{S}Z\right)}^{N}\left({p}_{1}{p}_{2}\right)\le max\left\{{\left(\overline{R}Y\right)}^{N}\left({p}_{1}\right),{\left(\overline{R}Y\right)}^{N}\left({p}_{2}\right)\right\}.$$

$\forall {p}_{1}{, p}_{2} \in P.$ In this instance, *Y* and *Z* are intuitionistic fuzzy rough subsets of $P \; and\; P\times P,$ respectively, while *R* and *S* are equivalence relations.

### Definition 2.2

The underlying intuitionistic fuzzy rough graph of $$\mathcal{G}=\left(\underline{\mathcal{G}},\overline{\mathcal{G}}\right)$$ have membership and non-membership is represented by $${\mathcal{G}}^{ \wedge } = \left( {\underline{{{\mathcal{G}}^{ \wedge } }} ,\overline{{{\mathcal{G}}^{ \wedge } }} } \right),$$ where $$\left( {\underline {R} Y} \right)^{ \wedge } = { }\left\{ {p_{1} \in P/\left( {\underline {R} Y} \right)\left( {p_{1} } \right) > 0} \right\},$$ $$\left( {\bar{R}Y} \right)^{ \wedge } =$$$$\left\{ {p_{1} \in P/\left( {\overline{R}Y} \right)\left( {p_{1} } \right) > 0} \right\}$$, $$\left( {\underline {S} Z} \right)^{ \wedge } = { }\left\{ {p_{1} p_{2} \in P \times P/\left( {\underline {S} Z} \right)\left( {p_{1} p_{2} } \right) > 0} \right\},$$ $$\left( {\bar{S}Z} \right)^{ \wedge } = \left\{ {p_{1} p_{2} \in P \times P/} \right.$$$$\left. {\left( {\bar{S}Z} \right)\left( {p_{1} p_{2} } \right) > 0} \right\}$$. Here $$\underline{{{\mathcal{G}}^{ \wedge } }} = \left( {\left( {\underline {R} Y} \right)^{ \wedge } ,\left( {\underline {S} Z} \right)^{ \wedge } } \right)$$ and $$\overline{{{\mathcal{G}}^{ \wedge } }} = \left( {\left( {\overline{R}Y} \right)^{ \wedge } ,\left( {\overline{S}Z} \right)^{ \wedge } } \right).$$ Thus, $$\underline{{{\mathcal{G}}^{ \wedge } }} {\text{ and }}\overline{{{\mathcal{G}}^{ \wedge } }}$$ are simply supports of the intuitionistic fuzzy rough graphs $$\underline{\mathcal{G}} \;and\; \overline{\mathcal{G}}$$.

### Definition 2.3

An IFRG $\widetilde{\mathcal{G}}=\left(\underline{\widetilde{\mathcal{G}}},\overline{\widetilde{\mathcal{G}}}\right)$ is referred to as a partial intuitionistic fuzzy rough subgraph of IFRG. $\mathcal{G}=\left(\underline{\mathcal{G}}, \overline{\mathcal{G}}\right)$ if $\left(\underline{R}\widetilde{Y}\right)\left({p}_{1}\right)\le \left(\underline{R}Y\right)\left({p}_{1}\right)$, $\left(\underline{S}\widetilde{Z}\right)\left({p}_{1},{p}_{2}\right)\le \left(\underline{S}Z\right)\left({p}_{1},{p}_{2}\right)$, $\left(\overline{R}\widetilde{Y}\right)\left({p}_{1}\right)\le \left(\overline{R}Y\right)\left({p}_{1}\right),$ $\left(\overline{S}\widetilde{Z}\right)\left({p}_{1},{p}_{2}\right)\le \left(\overline{S}Z\right)\left({p}_{1},{p}_{2}\right)$ $\forall$ ${p}_{1} \in \widetilde{Y}$ and $\left({p}_{1},{p}_{2}\right)\in \widetilde{Z}$.

### Definition 2.4

In $\mathcal{G}=\left(\underline{\mathcal{G}},\overline{\mathcal{G}}\right)$, let a path $P : {p}_{0}\to {p}_{1}\to {p}_{2}\to \cdots \to {p}_{n}$ of length *n* be the intuitionistic fuzzy rough path. If $P$ is the intuitionistic fuzzy rough path in $\underline{\mathcal{G}}\text{ likewise in }\overline{\mathcal{G}}$ of length *n* from ${w}_{0}$ to ${w}_{n}$. The least powerful edge in $\mathcal{G}=\left(\underline{\mathcal{G}},\overline{\mathcal{G}}\right)$ has the smallest membership and the largest non-membership value. The total degree of membership and non-membership of the least powerful edge in both $\underline{\mathcal{G}}$ and $\overline{\mathcal{G}}$ describe the route’s strength $P$. The connectedness strength from ${p}_{1}\; to\; {p}_{2}$ in $\mathcal{G}=\left(\underline{\mathcal{G}},\overline{\mathcal{G}}\right)$ is represented by the variable ${CONN}_{\mathcal{G}}({p}_{1}, {p}_{2})$, which is defined as the total of the greatest strengths of all paths from ${p}_{1}\; to \; {p}_{2}$ in $\mathcal{G}=\left(\underline{\mathcal{G}},\overline{\mathcal{G}}\right)$ If the strength of an intuitionistic fuzzy rough path in $\mathcal{G}=\left(\underline{\mathcal{G}},\overline{\mathcal{G}}\right)$ equals ${CONN}_{\mathcal{G}}({p}_{1}, {p}_{2}),$ then $P$ is regarded as the strongest ${p}_{1} to {p}_{2}$ intuitionistic fuzzy rough path (IFRP).

### Definition 2.5

In an IFRG in $\mathcal{G}=\left(\underline{\mathcal{G}},\overline{\mathcal{G}}\right)$, an edge from ${p}_{1} to {p}_{2}$ is classified as follows:

1. $\alpha$ – strong fuzzy edge in $\underline{\mathcal{G}}$ as well as in $\overline{\mathcal{G}}$ if

$${\left(\underline{S}Z\right)}^{M}\left({p}_{1},{p}_{2}\right)>{CONN}_{\underline{M\mathcal{G}}-{p}_{1}{p}_{2}}\left({p}_{1},{p}_{2}\right) and {\left(\underline{S}Z\right)}^{N}\left({p}_{1},{p}_{2}\right)<{CONN}_{\underline{N\mathcal{G}}-{p}_{1}{p}_{2}}\left({p}_{1},{p}_{2}\right),$$

$${\left(\overline{S}Z\right)}^{M}({p}_{1}, {p}_{2}) > {CONN}_{\overline{M\mathcal{G}}-{p}_{1}{p}_{2}} ({p}_{1}, {p}_{2}) and {\left(\overline{S}Z\right)}^{N}({p}_{1}, {p}_{2}) < {CONN}_{\overline{N\mathcal{G}}-{p}_{1}{p}_{2}} ({p}_{1}, {p}_{2}).$$

2. $\beta$ – strong fuzzy edge in $\underline{\mathcal{G}}$ as well as in $\overline{\mathcal{G}}$ if

$${\left(\underline{S}Z\right)}^{M}\left({p}_{1},{p}_{2}\right)={CONN}_{\underline{M\mathcal{G}}-{p}_{1}{p}_{2}}\left({p}_{1},{p}_{2}\right) and {\left(\underline{S}Z\right)}^{N}\left({p}_{1},{p}_{2}\right)={CONN}_{\underline{N\mathcal{G}}-{p}_{1}{p}_{2}}\left({p}_{1},{p}_{2}\right),$$

$${\left(\overline{S}Z\right)}^{M}({p}_{1}, {p}_{2}) = {CONN}_{\overline{M\mathcal{G}}-{p}_{1}{p}_{2}} ({p}_{1}, {p}_{2}) and {\left(\overline{S}Z\right)}^{N}({p}_{1}, {p}_{2}) = {CONN}_{\overline{N\mathcal{G}}-{p}_{1}{p}_{2}} ({p}_{1}, {p}_{2}).$$

3. $\delta$ – weak fuzzy edge in $\underline{\mathcal{G}}$ as well as in $\overline{\mathcal{G}}$ if

$${\left(\underline{S}Z\right)}^{M}\left({p}_{1},{p}_{2}\right)<{CONN}_{\underline{M\mathcal{G}}-{p}_{1}{p}_{2}}\left({p}_{1},{p}_{2}\right) and {\left(\underline{S}Z\right)}^{N}\left({p}_{1},{p}_{2}\right) > {CONN}_{\underline{N\mathcal{G}}-{p}_{1}{p}_{2}}\left({p}_{1},{p}_{2}\right),$$

$${\left(\overline{S}Z\right)}^{M}({p}_{1}, {p}_{2}) <{CONN}_{\overline{M\mathcal{G}}-{p}_{1}{p}_{2}} ({p}_{1}, {p}_{2}) and {\left(\overline{S}Z\right)}^{N}({p}_{1}, {p}_{2}) > {CONN}_{\overline{N\mathcal{G}}-{p}_{1}{p}_{2}} ({p}_{1}, {p}_{2}).$$

In $\mathcal{G}=\left(\underline{\mathcal{G}},\overline{\mathcal{G}}\right)$, an edge is considered strong intuitionistic fuzzy rough if it is either $\alpha$ – strong or $\beta$ – strong.

### Definition 2.6

If every edge on path $P$ is a strong intuitionistic fuzzy rough edge, then path $P$ is a strong intuitionistic fuzzy rough path.

### Definition 2.7

An intuitionistic fuzzy rough bridge of $\mathcal{G}=\left(\underline{\mathcal{G}},\overline{\mathcal{G}}\right)$ is defined as an edge ${p}_{1}{p}_{2} \in P \times P$ if the removal of an intuitionistic fuzzy rough edge ${p}_{1}{p}_{2}$ decreases the strength of connectivity between several pairs of vertices in $\mathcal{G}=\left(\underline{\mathcal{G}},\overline{\mathcal{G}}\right).$

### Definition 2.8

An IFRG without intuitionistic fuzzy rough-cut vertices is called an intuitionistic fuzzy rough block, or simply a block. Similarly, if the elimination of an intuitionistic fuzzy rough vertex ${p}_{1}$ decreases the strength of connectedness between several pairs of vertices in $\mathcal{G}=\left(\underline{\mathcal{G}},\overline{\mathcal{G}}\right)$, ${p}_{1}$ is called an intuitionistic fuzzy rough-cut vertex of $\mathcal{G}=\left(\underline{\mathcal{G}},\overline{\mathcal{G}}\right).$

### Definition 2.9

There is an isomorphism $b : {\mathcal{G}}_{1}\to {\mathcal{G}}_{2}$ when two IFRGs, ${\mathcal{G}}_{1}=(\underline{{\mathcal{G}}_{1}},\overline{{\mathcal{G}}_{1}})$ and ${\mathcal{G}}_{2}=(\underline{{\mathcal{G}}_{2}},\overline{{\mathcal{G}}_{2}})$, are considered. If $\underline{b} : \underline{{\mathcal{G}}_{1}}\to \underline{{\mathcal{G}}_{2}}$ and $\overline{b} : \overline{{\mathcal{G}}_{1}} \to \overline{{\mathcal{G}}_{2}}$ are two isomorphisms. i.e. there are two bijective mappings ($\underline{b}, \overline{b}) : P\to P$ where in.

$$\begin{aligned} ({\text{i}})\;\;\;\left( {\underline{R} Y_{1} } \right)^{M} (p_{1} ) = & \left( {\underline{R} Y_{2} } \right)^{M} (\underline{b} (p_{1} ))and\left( {\underline{R} Y_{1} } \right)^{N} (p_{1} ) = \left( {\underline{R} Y_{2} } \right)^{N} (\underline{b} (p_{1} )), \\ \left( {\underline{S} Z_{1} } \right)^{M} (p_{1} p_{2} ) = & \left( {\underline{S} Z_{2} } \right)^{M} (\underline{b} (p_{1} )\underline{b} (p_{2} ))and\left( {\underline{S} Z_{1} } \right)^{N} (p_{1} p_{2} ) = \left( {\underline{S} Z_{2} } \right)^{N} (\underline{b} (p_{1} )\underline{b} (p_{2} )),\forall p_{1} \in P,p_{1} p_{2} \in P \times P. \\ \end{aligned}$$

$$\begin{aligned} ({\text{ii}})\;\;\;\left( {\bar{R}Y_{1} } \right)^{M} (p_{1} ) = & \left( {\bar{R}Y_{2} } \right)^{M} (\bar{b}(p_{1} ))and\left( {\bar{R}Y_{1} } \right)^{N} (p_{1} ) = \left( {\bar{R}Y_{2} } \right)^{N} (\bar{b}(p_{1} )), \\ \left( {\bar{S}Z_{1} } \right)^{M} (p_{1} p_{2} ) = & \left( {\bar{S}Z_{2} } \right)^{M} (\bar{b}(p_{1} )\bar{b}(p_{2} ))and\left( {\bar{S}Z_{1} } \right)^{N} (p_{1} p_{2} ) = \left( {\bar{S}Z_{2} } \right)^{N} (\bar{b}(p_{1} )\bar{b}(p_{2} )),\forall p_{1} \in P,p_{1} p_{2} \in P \times P. \\ \end{aligned}$$

### Definition 2.10

Consider an IFRG $\mathcal{G}=\left(\underline{\mathcal{G}},\overline{\mathcal{G}}\right)$. A strong intuitionistic fuzzy rough path *P* from ${p}_{1} to {p}_{n}$ is said to be an intuitionistic fuzzy rough geodesic in $\mathcal{G}$. In both $\underline{G},\overline{G}$, that is, there is not a stronger route P that is shorter from vertex ${p}_{1}$ to vertex ${p}_{n}$. The total of all intuitionistic fuzzy rough edges’ membership and non-membership degrees within a geodesic is called a geodesic value.

### Example 2.11

**Fig. 1:**
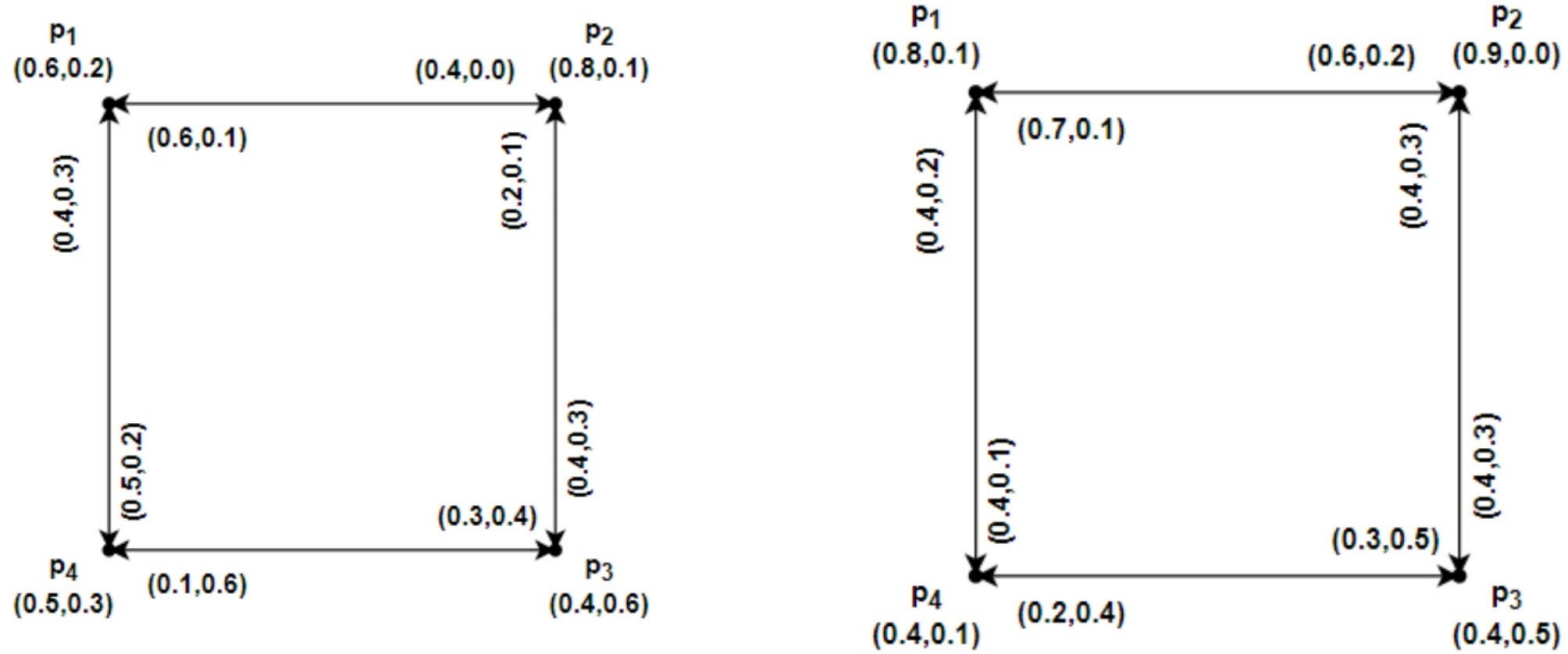
$$\mathcal{G}=\left(\underline{\mathcal{G}},\overline{\mathcal{G}}\right)$$ the IFRG.

Let us consider an IFRG $\mathcal{G}=\left(\underline{\mathcal{G}},\overline{\mathcal{G}}\right)$ given in Fig. 1. The following describes the strength of connectivity between vertices of IFRG for edges:

$${CONN}_{\underline{\mathcal{G}}-\left({p}_{1},{p}_{2}\right)}\left({p}_{1},{p}_{2}\right)=\left(\text{0.2,0.4}\right), { CONN}_{\underline{\mathcal{G}}-\left({p}_{2},{p}_{1}\right)}\left({p}_{2},{p}_{1}\right)=\left(\text{0.1,0.6}\right),$$

$${CONN}_{\overline{\mathcal{G}}-\left({p}_{1},{p}_{2}\right)}\left({p}_{1},{p}_{2}\right)=\left(\text{0.3,0.5}\right),{ CONN}_{\overline{\mathcal{G}}-({p}_{2},{p}_{1})}({p}_{2},{p}_{1})=(\text{0.2,0.4}),$$

$${CONN}_{\underline{\mathcal{G}}-({p}_{1},{p}_{3})}({p}_{1},{p}_{3})=(\text{0.4,0.3}), {CONN}_{\underline{\mathcal{G}}-({p}_{3},{p}_{1})}({p}_{3},{p}_{1})=(\text{0.2,0.1}),$$

$${CONN}_{\overline{\mathcal{G}}-({p}_{1},{p}_{3})}({p}_{1},{p}_{3})=(\text{0.4,0.3}), {CONN}_{\overline{\mathcal{G}}-({p}_{3},{p}_{1})}({p}_{3},{p}_{1})=(\text{0.4,0.3}),$$

$${CONN}_{\underline{\mathcal{G}}-({p}_{1},{p}_{4})}({p}_{1},{p}_{4})=(\text{0.1,0.6}), {CONN}_{\underline{\mathcal{G}}-({p}_{4},{p}_{1})}({p}_{4},{p}_{1})=(\text{0.2,0.4}),$$

$${CONN}_{\overline{\mathcal{G}}-({p}_{1},{p}_{4})}({p}_{1},{p}_{4})=(\text{0.2,0.4}), {CONN}_{\overline{\mathcal{G}}-({p}_{4},{p}_{1})}({p}_{4},{p}_{1})=(\text{0.3,0.5}),$$

$${CONN}_{\underline{\mathcal{G}}-({p}_{2},{p}_{3})}({p}_{2},{p}_{3})=(\text{0.3,0.4}), {CONN}_{\underline{\mathcal{G}}-({p}_{3},{p}_{2})}({p}_{3},{p}_{2})=(\text{0.1,0.6}),$$

$${CONN}_{\overline{\mathcal{G}}-({p}_{2},{p}_{3})}({p}_{2},{p}_{3})=(\text{0.3,0.5}), {CONN}_{\overline{\mathcal{G}}-({p}_{3},{p}_{2})}({p}_{3},{p}_{2})=(\text{0.2,0.4}),$$

$${CONN}_{\underline{\mathcal{G}}-({p}_{2},{p}_{4})}({p}_{2},{p}_{4})=(\text{0.5,0.2}), {CONN}_{\underline{\mathcal{G}}-({p}_{4},{p}_{2})}({p}_{4},{p}_{2})=(\text{0.4,0.3}),$$

$${CONN}_{\overline{\mathcal{G}}-({p}_{2},{p}_{4})}({p}_{2},{p}_{4})=(\text{0.4,0.1}), {CONN}_{\overline{\mathcal{G}}-({p}_{4},{p}_{2})}({p}_{4},{p}_{2})=(\text{0.4,0.2}),$$

$${CONN}_{\underline{\mathcal{G}}-({p}_{3},{p}_{4})}({p}_{3},{p}_{4})=(\text{0.2,0.2}), {CONN}_{\underline{\mathcal{G}}-({p}_{4},{p}_{3})}({p}_{4},{p}_{3})=(\text{0.4,0.3}),$$

$${CONN}_{\overline{\mathcal{G}}-({p}_{3},{p}_{4})}({p}_{3},{p}_{4})=(\text{0.4,0.3}), {CONN}_{\overline{\mathcal{G}}-({p}_{4},{p}_{3})}({p}_{4},{p}_{3})=(\text{0.4,0.3}).$$

Confirming that the edges are ${p}_{1}{p}_{2},$ ${p}_{2}{p}_{1},$ ${p}_{2}{p}_{3},$ ${p}_{3}{p}_{2},$ ${p}_{1}{p}_{4},$ ${p}_{4}{p}_{1}$ are $\alpha$ – strong intuitionistic fuzzy rough edges in $\underline{\mathcal{G}}$ and ${p}_{1}{p}_{2},$ ${p}_{2}{p}_{1},$ ${p}_{2}{p}_{3},$ ${p}_{3}{p}_{2},$ ${p}_{1}{p}_{4},$ ${p}_{4}{p}_{1}$ are $\alpha$ – strong intuitionistic fuzzy rough edges in $\overline{\mathcal{G}}$, respectively. Therefore, the edges ${p}_{1}{p}_{2},$ ${p}_{2}{p}_{1},$ ${p}_{2}{p}_{3},$ ${p}_{3}{p}_{2},$ ${p}_{1}{p}_{4},$ ${p}_{4}{p}_{1}$ are $\alpha$ – strong intuitionistic fuzzy rough edges in $\mathcal{G}=\left(\underline{\mathcal{G}},\overline{\mathcal{G}}\right).$ Similarly ${p}_{3}{p}_{4},$ ${p}_{4}{p}_{3}$ are $\beta$-strong intuitionistic fuzzy rough edges in $\mathcal{G}=\left(\underline{\mathcal{G}},\overline{\mathcal{G}}\right)$. So ${p}_{1}{p}_{2},$ ${p}_{2}{p}_{1},$ ${p}_{2}{p}_{3},$ ${p}_{3}{p}_{2},$ ${p}_{1}{p}_{4},$ ${p}_{4}{p}_{1}$, ${p}_{3}{p}_{4},$ ${p}_{4}{p}_{3}$ are robust intuitionistic fuzzy rough edges of $\mathcal{G}=\left(\underline{\mathcal{G}},\overline{\mathcal{G}}\right)$.

All intuitionistic fuzzy rough edges in this case are strong, with the exception of $${p}_{3}{p}_{4},$$ $${p}_{4}{p}_{3}$$. Therefore, $${p}_{3}\to {p}_{2}\to {p}_{1}\to {p}_{4}$$ , $${p}_{4}\to {p}_{1}\to {p}_{2}\to {p}_{3}$$ is the only strong, shorter route that connects $${p}_{3}{ to p}_{4} and$$ $${p}_{4} to {p}_{3}$$ in both $$\underline{\mathcal{G}} and \overline{\mathcal{G}}$$. As a result, the geodesic that connects the vertex is the directed path $${p}_{3}{ to p}_{4} and$$ $${p}_{4} to {p}_{3}$$ in $$\underline{\mathcal{G}} as well as in \overline{\mathcal{G}}$$ and values of geodesic in $$\underline{\mathcal{G}} and \overline{\mathcal{G}}$$ are (1.3, 0.4), (1.5, 0.5) and (1.2,0.6), (1.4,0.7) respectively.

### Definition 2.12

Consider an IFRG $\mathcal{G}=\left(\underline{\mathcal{G}},\overline{\mathcal{G}}\right)$. The connectivity index $\left(\mathcal{C}\mathcal{I}\right)$ of $\mathcal{G}=\left(\underline{\mathcal{G}},\overline{\mathcal{G}}\right) is$ defined as:

$$\mathcal{C}\mathcal{I}(\mathcal{G}) = \mathcal{C}\mathcal{I}(\underline{\mathcal{G}})+\mathcal{C}\mathcal{I}(\overline{\mathcal{G}}),$$

where

$$\mathcal{C}\mathcal{I}(\underline{\mathcal{G}})=\sum_{{p}_{1},{p}_{2}\in V(\underline{\mathcal{G}})}(\underline{M})({p}_{1})(\underline{M})({p}_{2}){CONN}_{\underline{M\mathcal{G}}}({p}_{1},{p}_{2})+\sum_{{p}_{1},{p}_{2}\in V(\underline{\mathcal{G}})}(\underline{N})({p}_{1})(\underline{N})({p}_{2}){CONN}_{\underline{N\mathcal{G}}}({p}_{1},{p}_{2}),$$

$$\mathcal{C}\mathcal{I}(\overline{\mathcal{G}})=\sum_{{p}_{1},{p}_{2}\in V(\overline{\mathcal{G}})}(\overline{M})({p}_{1})(\overline{M})({p}_{2}){CONN}_{\overline{M\mathcal{G}}}({p}_{1},{p}_{2})+\sum_{{p}_{1},{p}_{2}\in V(\overline{\mathcal{G}})}(\overline{N})({p}_{1})(\overline{N})({p}_{2}){CONN}_{\overline{N\mathcal{G}}}({p}_{1},{p}_{2}).$$

Note that ${CONN}_{\underline{M\mathcal{G}}}({p}_{1},{p}_{2})$,${CONN}_{\underline{N\mathcal{G}}}({p}_{1},{p}_{2}), {CONN}_{\overline{M\mathcal{G}}}({p}_{1},{p}_{2})$ and ${CONN}_{\overline{N\mathcal{G}}}({p}_{1},{p}_{2})$ is the strength of connection ($\mathcal{S}\mathcal{C}$) between ${p}_{1} and {p}_{2}$ in $\underline{\mathcal{G}}$ and $\overline{\mathcal{G}}$ respectively.

### Definition 2.13

An IFRG $\mathcal{G}=\left(\underline{\mathcal{G}},\overline{\mathcal{G}}\right)$ should be considered. For $\mathcal{G}=\left(\underline{\mathcal{G}},\overline{\mathcal{G}}\right)$, the average connectivity index $\left(\mathcal{A}\mathcal{C}\mathcal{I}\right)$ is defined as follows:

$$\mathcal{A}\mathcal{C}\mathcal{I}(\mathcal{G})=\mathcal{A}\mathcal{C}\mathcal{I}(\underline{\mathcal{G}})+\mathcal{A}\mathcal{C}\mathcal{I}(\overline{\mathcal{G}}),$$

where

$$\mathcal{A}\mathcal{C}\mathcal{I}(\underline{\mathcal{G}})=\frac{1}{\left(\genfrac{}{}{0pt}{}{n}{2}\right)}\sum_{{p}_{1},{p}_{2}\in V(\underline{\mathcal{G}})}(\underline{M})({p}_{1})(\underline{M})({p}_{2}){CONN}_{\underline{M\mathcal{G}}}({p}_{1},{p}_{2})+\frac{1}{\left(\genfrac{}{}{0pt}{}{n}{2}\right)}\sum_{{p}_{1},{p}_{2}\in V(\underline{\mathcal{G}})}(\underline{N})({p}_{1})(\underline{N})({p}_{2}){CONN}_{\underline{N\mathcal{G}}}({p}_{1},{p}_{2}),$$

$$\mathcal{A}\mathcal{C}\mathcal{I}(\overline{\mathcal{G}})=\frac{1}{\left(\genfrac{}{}{0pt}{}{n}{2}\right)}\sum_{{p}_{1},{p}_{2}\in V(\overline{\mathcal{G}})}(\overline{M})({p}_{1})(\overline{M})({p}_{2}){CONN}_{\overline{M\mathcal{G}}}({p}_{1},{p}_{2})+\frac{1}{\left(\genfrac{}{}{0pt}{}{n}{2}\right)}\sum_{{p}_{1},{p}_{2}\in V(\overline{\mathcal{G}})}(\overline{N})({p}_{1})(\overline{N})({p}_{2}){CONN}_{\overline{N\mathcal{G}}}({p}_{1},{p}_{2}).$$

In this case, *n* stands for all of $\mathcal{G}$ 's the total number of vertices.

### Example 2.14

**Fig. 2:**
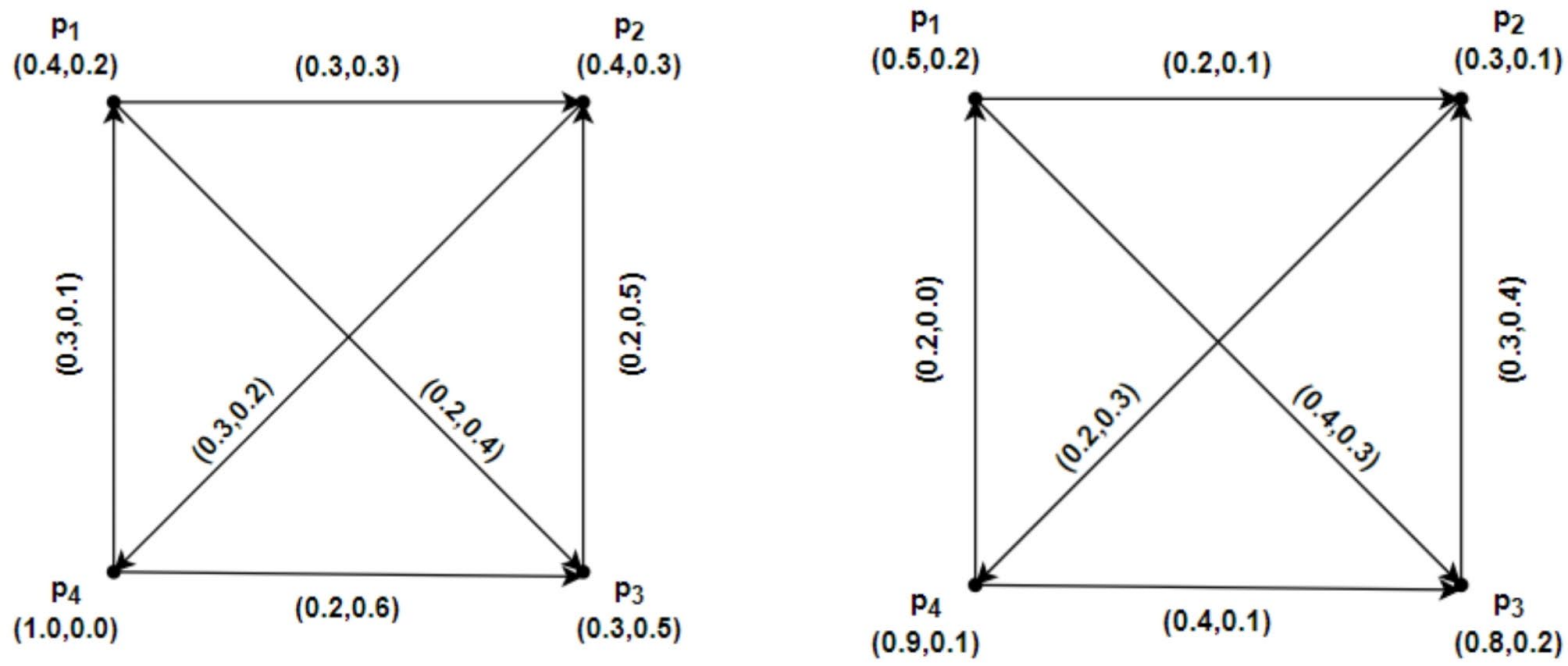
$$\mathcal{G}=\left(\underline{\mathcal{G}},\overline{\mathcal{G}}\right)$$ the IFRG.

An IFRG $\mathcal{G}=\left(\underline{\mathcal{G}},\overline{\mathcal{G}}\right)$ on $u=\left\{{p}_{1}, {p}_{2},{p}_{3},{p}_{4}\right\}$ in Fig. 2. is considered. By using direct calculations, we demonstrate IFRGs. The $\mathcal{S}\mathcal{C}$ between an IFRG’s vertices is as follows:

$${CONN}_{\underline{\mathcal{G}}({p}_{1},{p}_{2})}({p}_{1},{p}_{2})=(\text{0.3,0.3}), { CONN}_{\underline{\mathcal{G}}({p}_{2},{p}_{1})}({p}_{2},{p}_{1})=(\text{0.3,0.2}),$$

$${CONN}_{\overline{\mathcal{G}}({p}_{1},{p}_{2})}({p}_{1},{p}_{2})=(\text{0.3,0.1}), { CONN}_{\overline{\mathcal{G}}({p}_{2},{p}_{1})}({p}_{2},{p}_{1})=(\text{0.2,0.3}),$$

$${CONN}_{\underline{\mathcal{G}}({p}_{1},{p}_{3})}({p}_{1},{p}_{3})=(\text{0.2,0.4}), {CONN}_{\underline{\mathcal{G}}({p}_{3},{p}_{1})}({p}_{3},{p}_{1})=(\text{0.2,0.5}),$$

$${CONN}_{\overline{\mathcal{G}}({p}_{1},{p}_{3})}({p}_{1},{p}_{3})=(\text{0.4,0.3}), {CONN}_{\overline{\mathcal{G}}({p}_{3},{p}_{1})}({p}_{3},{p}_{1})=(\text{0.2,0.4}),$$

$${CONN}_{\underline{\mathcal{G}}({p}_{1},{p}_{4})}({p}_{1},{p}_{4})=(\text{0.3,0.3}), {CONN}_{\underline{\mathcal{G}}({p}_{4},{p}_{1})}({p}_{4},{p}_{1})=(\text{0.3,0.1}),$$

$${CONN}_{\overline{\mathcal{G}}({p}_{1},{p}_{4})}({p}_{1},{p}_{4})=(\text{0.2,0.3}), {CONN}_{\overline{\mathcal{G}}({p}_{4},{p}_{1})}({p}_{4},{p}_{1})=(\text{0.2,0.0}),$$

$${CONN}_{\underline{\mathcal{G}}({p}_{2},{p}_{3})}({p}_{2},{p}_{3})=(\text{0.2,0.6}), {CONN}_{\underline{\mathcal{G}}({p}_{3},{p}_{2})}({p}_{3},{p}_{2})=(\text{0.2,0.5}),$$

$${CONN}_{\overline{\mathcal{G}}({p}_{2},{p}_{3})}({p}_{2},{p}_{3})=(\text{0.2,0.3}), {CONN}_{\overline{\mathcal{G}}({p}_{3},{p}_{2})}({p}_{3},{p}_{2})=(\text{0.3,0.4}),$$

$${CONN}_{\underline{\mathcal{G}}({p}_{2},{p}_{4})}({p}_{2},{p}_{4})=(\text{0.3,0.2}), {CONN}_{\underline{\mathcal{G}}({p}_{4},{p}_{2})}({p}_{4},{p}_{2})=(\text{0.3,0.3}),$$

$${CONN}_{\overline{\mathcal{G}}({p}_{2},{p}_{4})}({p}_{2},{p}_{4})=(\text{0.2,0.3}), {CONN}_{\overline{\mathcal{G}}({p}_{4},{p}_{2})}({p}_{4},{p}_{2})=(\text{0.3,0.1}),$$

$${CONN}_{\underline{\mathcal{G}}({p}_{3},{p}_{4})}({p}_{3},{p}_{4})=(\text{0.2,0.5}), {CONN}_{\underline{\mathcal{G}}({p}_{4},{p}_{3})}({p}_{4},{p}_{3})=(\text{0.2,0.4}),$$

$${CONN}_{\overline{\mathcal{G}}({p}_{3},{p}_{4})}({p}_{3},{p}_{4})=(\text{0.2,0.4}), {CONN}_{\overline{\mathcal{G}}({p}_{4},{p}_{3})}({p}_{4},{p}_{3})=(\text{0.4,0.1}).$$

As a result, the $\mathcal{C}\mathcal{I}$ and $\mathcal{A}\mathcal{C}\mathcal{I}$ of $\mathcal{G}=\left(\underline{\mathcal{G}},\overline{\mathcal{G}}\right)$ are as follows:

$$M\mathcal{C}\mathcal{I}(\underline{\mathcal{G}})=0.7920, N\mathcal{C}\mathcal{I}(\underline{\mathcal{G}})=0.2850, \mathcal{C}\mathcal{I}(\underline{\mathcal{G}})=1.0770.$$

$$M\mathcal{C}\mathcal{I}(\overline{\mathcal{G}})=1.1820, N\mathcal{C}\mathcal{I}(\overline{\mathcal{G}})=0.0700, \mathcal{C}\mathcal{I}(\overline{\mathcal{G}})=1.2520.$$

$$\mathcal{C}\mathcal{I}(\mathcal{G})=\mathcal{C}\mathcal{I}(\underline{\mathcal{G}})+\mathcal{C}\mathcal{I}(\overline{\mathcal{G}})=2.3290.$$

$$M\mathcal{A}\mathcal{C}\mathcal{I} (\underline{\mathcal{G}})= 0.1320, N\mathcal{A}\mathcal{C}\mathcal{I} (\underline{\mathcal{G}})=0.0475 , \mathcal{A}\mathcal{C}\mathcal{I} (\underline{\mathcal{G}})=0.1795 .$$

$$M\mathcal{A}\mathcal{C}\mathcal{I} (\overline{\mathcal{G}})=0.1970, N\mathcal{A}\mathcal{C}\mathcal{I} (\overline{\mathcal{G}})=0.0117 , \mathcal{A}\mathcal{C}\mathcal{I} (\overline{\mathcal{G}})=0.2087 .$$

$$\mathcal{A}\mathcal{C}\mathcal{I}(\mathcal{G})=\mathcal{A}\mathcal{C}\mathcal{I}(\underline{\mathcal{G}})+\mathcal{A}\mathcal{C}\mathcal{I}(\overline{\mathcal{G}})=0.3882.$$

IFGs contain valuable information by considering membership, non-membership, and hesitation degrees, providing a more comprehensive representation of uncertainty compared to FGs. However, there are scenarios where we require additional granularity to handle imprecision and boundary regions more effectively. In such cases, IFRGs are more suitable as they combine the strengths of intuitionistic fuzzy sets and rough sets, offering both membership and non-membership approximations. To meet these needs, we aim to extend the concept of $\mathcal{W}\mathcal{I}$ to IFRGs. This approach allows us to evaluate $\mathcal{W}\mathcal{I}$ in terms of both lower and upper approximations for membership and non-membership values. An interesting observation is that we derive four indices: two for lower approximations and two for upper approximations of membership and non-membership. For a consolidated measure, we aggregate these indices to provide a comprehensive single value, reflecting the enhanced information and precision offered by IFRGs.

## Wiener index of an intuitionistic fuzzy rough graph

The idea of the wiener index for intuitionistic fuzzy rough graphs was presented in this section. Graph invariants, or practically topological indices, have long been used to build molecules with the necessary properties, for example, in drug design. One such topological index that is employed in many fields, such as medical, facility location, communication, and cryptology, is the wiener index. A remarkable number of scenarios have been simulated by an IFRG. The standard interpretation for estimating the wiener index of an intuitionistic fuzzy rough graph is then given.

### Definition 3.1

Let us consider an IFRG $\mathcal{G}=\left(\underline{\mathcal{G}},\overline{\mathcal{G}}\right)$. The wiener index ($\mathcal{W}\mathcal{I})$ of $\mathcal{G}$ is defined by:

For lower approximation of IFRG:

$$\begin{aligned} {\mathcal{W}\mathcal{I}}(\underline{{\mathcal{G}}} ) = & \sum\limits_{{p_{1} ,p_{2} \in V(\underline{{\mathcal{G}}} )}} {\left( {(\underline{M} )(p_{1} ),(\underline{N} )(p_{1} )} \right)} \left( {(\underline{M} )(p_{2} ),(\underline{N} )(p_{2} )} \right)\left( {ds_{{\underline{{\mathcal{G}}} }} (p_{1} ,p_{2} )} \right), \\ = & \sum\limits_{{p_{1} ,p_{2} \in V(\underline{{\mathcal{G}}} )}} {\left( {(\underline{M} )(p_{1} ),(\underline{N} )(p_{1} )} \right)} \left( {(\underline{M} )(p_{2} ),(\underline{N} )(p_{2} )} \right)\left( {ds_{{\underline{{M{\mathcal{G}}}} }} (p_{1} ,p_{2} ),ds_{{\underline{{N{\mathcal{G}}}} }} (p_{1} ,p_{2} )} \right), \\ = & \sum\limits_{{p_{1} ,p_{2} \in V(\underline{{\mathcal{G}}} )}} {\left( {(\underline{M} )(p_{1} )(\underline{M} )(p_{2} )ds_{{\underline{{M{\mathcal{G}}}} }} (p_{1} ,p_{2} ) + (\underline{N} )(p_{1} )(\underline{N} )(p_{2} )ds_{{\underline{{N{\mathcal{G}}}} }} (p_{1} ,p_{2} )} \right)} , \\ {\mathcal{W}\mathcal{I}}(\underline{{\mathcal{G}}} ) = & \sum\limits_{{p_{1} ,p_{2} \in V(\underline{{\mathcal{G}}} )}} {(\underline{M} )} (p_{1} )(\underline{M} )(p_{2} )ds_{{\underline{{M{\mathcal{G}}}} }} (p_{1} ,p_{2} ) + \sum\limits_{{p_{1} ,p_{2} \in V(\underline{{\mathcal{G}}} )}} {(\underline{N} )} (p_{1} )(\underline{N} )(p_{2} )ds_{{\underline{{N{\mathcal{G}}}} }} (p_{1} ,p_{2} ), \\ \end{aligned}$$

For upper approximation of IFRG:

$$\begin{aligned} {\mathcal{W}\mathcal{I}}(\overline{{\mathcal{G}}} ) = & \sum\limits_{{p_{1} ,p_{2} \in V(\overline{{\mathcal{G}}} )}} {\left( {(\bar{M})(p_{1} ),(\bar{N})(p_{1} )} \right)} \left( {(\bar{M})(p_{2} ),(\bar{N})(p_{2} )} \right)\left( {ds_{{\overline{{\mathcal{G}}} }} (p_{1} ,p_{2} )} \right), \\ = & \sum\limits_{{p_{1} ,p_{2} \in V(\overline{{\mathcal{G}}} )}} {\left( {(\bar{M})(p_{1} ),(\bar{N})(p_{1} )} \right)} \left( {(\bar{M})(p_{2} ),(\bar{N})(p_{2} )} \right)\left( {ds_{{\overline{{M{\mathcal{G}}}} }} (p_{1} ,p_{2} ),ds_{{\overline{{N{\mathcal{G}}}} }} (p_{1} ,p_{2} )} \right), \\ = & \sum\limits_{{p_{1} ,p_{2} \in V(\overline{{\mathcal{G}}} )}} {\left( {(\bar{M})(p_{1} )(\bar{M})(p_{2} )ds_{{\overline{{M{\mathcal{G}}}} }} (p_{1} ,p_{2} ) + (\bar{N})(p_{1} )(\bar{N})(p_{2} )ds_{{\overline{{N{\mathcal{G}}}} }} (p_{1} ,p_{2} )} \right)} , \\ {\mathcal{W}\mathcal{I}}(\overline{{\mathcal{G}}} ) = & \sum\limits_{{p_{1} ,p_{2} \in V(\overline{{\mathcal{G}}} )}} {(\bar{M})} (p_{1} )(\bar{M})(p_{2} )ds_{{\overline{{M{\mathcal{G}}}} }} (p_{1} ,p_{2} ) + \sum\limits_{{p_{1} ,p_{2} \in V(\overline{{\mathcal{G}}} )}} {(\bar{N})} (p_{1} )(\bar{N})(p_{2} )ds_{{\overline{{N{\mathcal{G}}}} }} (p_{1} ,p_{2} ). \\ {\mathcal{W}\mathcal{I}}({\mathcal{G}}) = & {\mathcal{W}\mathcal{I}}(\underline{{\mathcal{G}}} ) + {\mathcal{W}\mathcal{I}}(\overline{{\mathcal{G}}} ), \\ \end{aligned}$$

Here ${ds}_{\underline{\mathcal{G}}}({p}_{1},{p}_{2})$ and ${ds}_{\overline{\mathcal{G}}}({p}_{1},{p}_{2})$ depict values of those geodesics from vertex ${p}_{1}$ to vertex ${p}_{2}$ whose total is minimum in $\underline{\mathcal{G}}$ as well as in $\overline{\mathcal{G}}$, respectively.

### Example 3.2

**Fig. 3:**
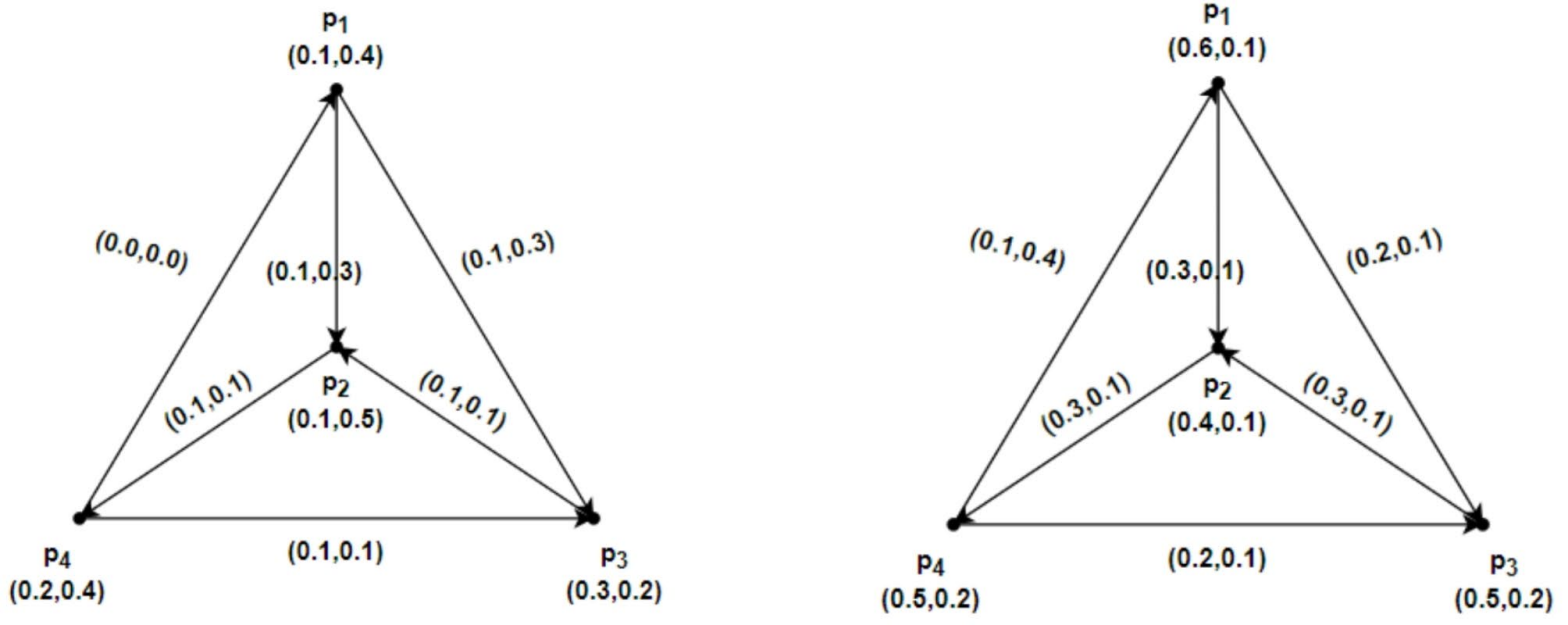
$$\mathcal{G}=\left(\underline{\mathcal{G}},\overline{\mathcal{G}}\right)$$ the IFRG.

Assume that $u=\left\{{p}_{1}, {p}_{2},{p}_{3},{p}_{4}\right\}$ has an IFRG $\mathcal{G}=\left(\underline{\mathcal{G}},\overline{\mathcal{G}}\right)$. Accordingly, IFRG is shown in Fig. 3 by $\underline{\mathcal{G}}=\left(\underline{R}Y,\underline{S}Z\right)$ and $\overline{\mathcal{G}}=\left(\overline{R}Y,\overline{S}Z\right)$. The weight of the geodesic connecting the IFRG vertices whose total is the smallest may be calculated directly as follows:

$${ds}_{\underline{\mathcal{G}}}({p}_{1},{p}_{2})=(\text{0.1,0.3}),{ds}_{\underline{\mathcal{G}}}({p}_{2},{p}_{1})=(\text{0.1,0.1}),{ds}_{\overline{\mathcal{G}}}({p}_{1},{p}_{2})=(\text{0.3,0.1}), {ds}_{\overline{\mathcal{G}}}({p}_{2},{p}_{1})=(\text{0.4,0.5}),$$

$${ds}_{\underline{\mathcal{G}}}({p}_{1},{p}_{3})=(\text{0.1,0.3}), {ds}_{\underline{\mathcal{G}}}({p}_{3},{p}_{1})=(\text{0.2,0.2}),{ds}_{\overline{\mathcal{G}}}({p}_{1},{p}_{3})=(\text{0.2,0.1}), {ds}_{\overline{\mathcal{G}}}({p}_{3},{p}_{1})=(\text{0.7,0.6}),$$

$${ds}_{\underline{\mathcal{G}}}({p}_{1},{p}_{4})=(\text{0.2,0.4}), {ds}_{\underline{\mathcal{G}}}({p}_{4},{p}_{1})=(\text{0.0,0.0}),{ds}_{\overline{\mathcal{G}}}({p}_{1},{p}_{4})=(\text{0.4,0.2}), {ds}_{\overline{\mathcal{G}}}({p}_{4},{p}_{1})=(\text{0.1,0.4}),$$

$${ds}_{\underline{\mathcal{G}}}({p}_{2},{p}_{3})=(\text{0.2,0.2}), {ds}_{\underline{\mathcal{G}}}({p}_{3},{p}_{2})=(\text{0.1,0.1}),{ds}_{\overline{\mathcal{G}}}({p}_{2},{p}_{3})=(\text{0.5,0.2}), {ds}_{\overline{\mathcal{G}}}({p}_{3},{p}_{2})=(\text{0.3,0.1}),$$

$${ds}_{\underline{\mathcal{G}}}({p}_{2},{p}_{4})=(\text{0.1,0.1}), {ds}_{\underline{\mathcal{G}}}({p}_{4},{p}_{2})=(\text{0.1,0.2}),{ds}_{\overline{\mathcal{G}}}({p}_{2},{p}_{4})=(\text{0.3,0.1}), {ds}_{\overline{\mathcal{G}}}({p}_{4},{p}_{2})=(\text{0.4,0.2}),$$

$${ds}_{\underline{\mathcal{G}}}({p}_{3},{p}_{4})=(\text{0.2,0.2}), {ds}_{\underline{\mathcal{G}}}({p}_{4},{p}_{3})=(\text{0.1,0.1}),{ds}_{\overline{\mathcal{G}}}({p}_{3},{p}_{4})=(\text{0.6,0.2}), {ds}_{\overline{\mathcal{G}}}({p}_{4},{p}_{3})=(\text{0.2,0.1}).$$

By above computations and from Fig. 3, we have

$$\mathcal{W}\mathcal{I}(\underline{\mathcal{G}})=0.3440, \mathcal{W}\mathcal{I}(\overline{\mathcal{G}})=1.2440, and \mathcal{W}\mathcal{I}(\mathcal{G})=1.5880.$$

*Note*: If we assume that $\mathcal{G}=\left(\underline{\mathcal{G}},\overline{\mathcal{G}}\right)$ an IFRG and $\mathcal{H}=\left(\underline{\mathcal{H}} ,\overline{\mathcal{H}}\right)$ is the partial intuitionistic fuzzy rough subgraph of $\mathcal{G}=\left(\underline{\mathcal{G}},\overline{\mathcal{G}}\right)$. In this case, $\mathcal{W}\mathcal{I}(\mathcal{H})$ need not necessarily be less than or equal to $\mathcal{W}\mathcal{I}(\mathcal{G})$. This is seen in the example that follows.

### Example 3.3

**Fig. 4:**
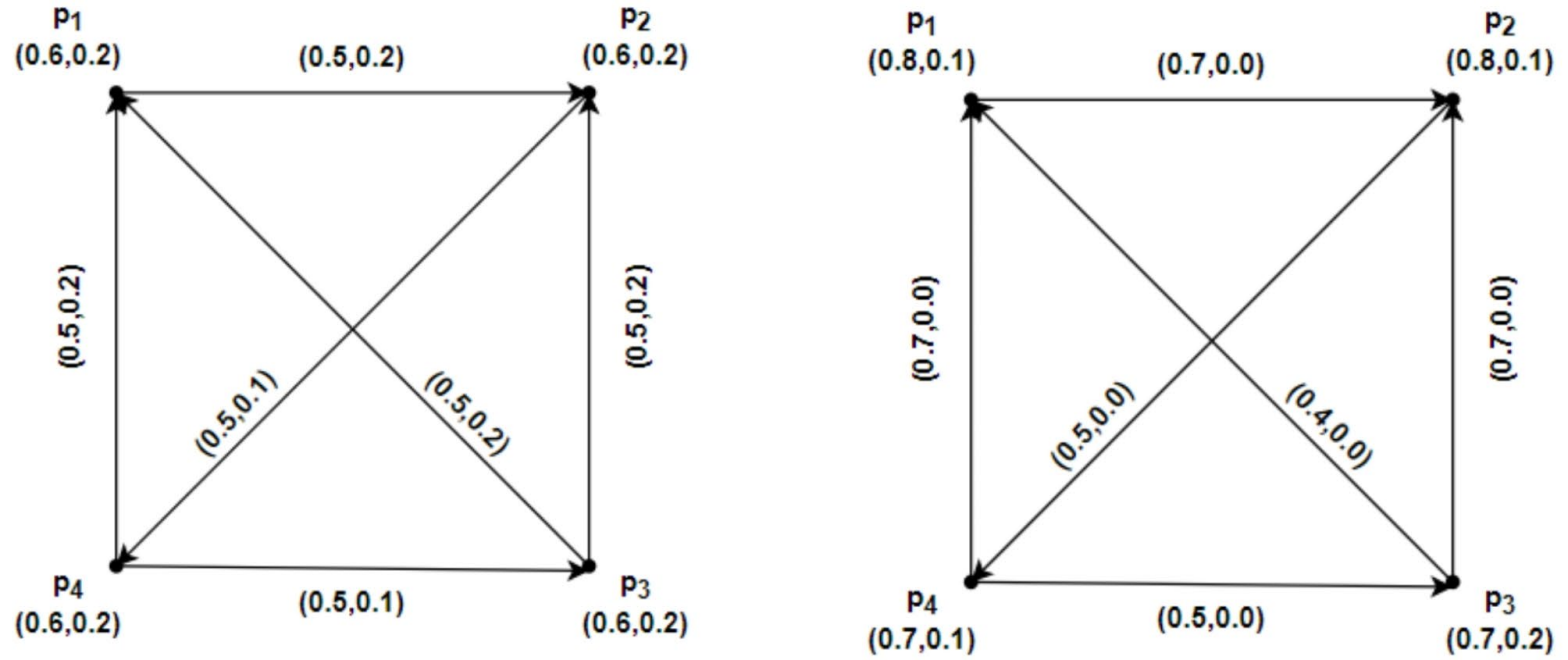
$\mathcal{G}=\left(\underline{\mathcal{G}},\overline{\mathcal{G}}\right)$ the IFRG with $\mathcal{W}\mathcal{I}(\mathcal{G})>\mathcal{W}\mathcal{I}(\mathcal{H})$.

**Fig. 5:**
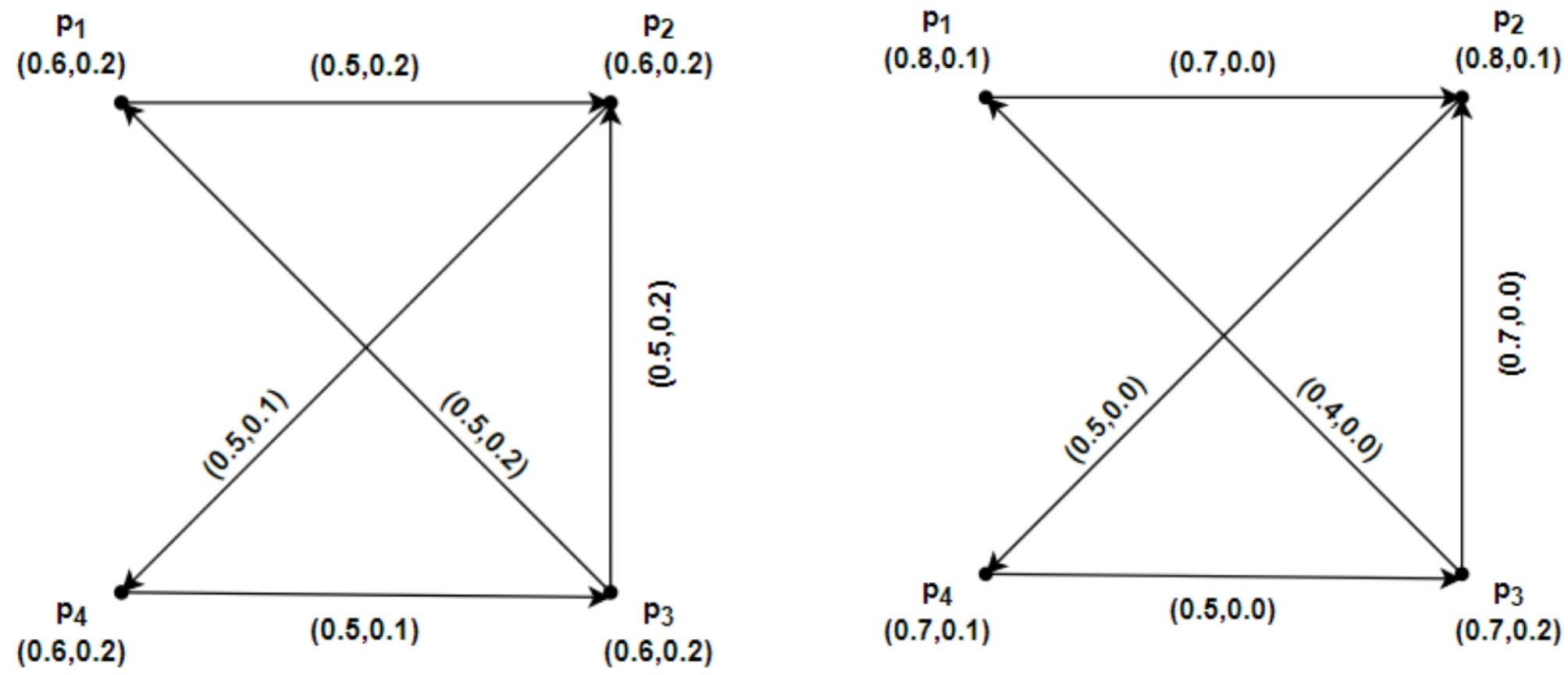
$\mathcal{H}=\left(\underline{\mathcal{H}},\overline{\mathcal{H}}\right)$ the partial IFR-subgraph of $\mathcal{G}=\left(\underline{\mathcal{G}},\overline{\mathcal{G}}\right)$.

Consider $\mathcal{G}=\left(\underline{\mathcal{G}},\overline{\mathcal{G}}\right)$ be an IFRG on $u=\left\{{p}_{1}, {p}_{2},{p}_{3},{p}_{4}\right\}$ and $\mathcal{H}=\left(\underline{\mathcal{H}} ,\overline{\mathcal{H}}\right)$ a partial intuitionistic fuzzy rough subgraph of $\mathcal{G}=\left(\underline{\mathcal{G}},\overline{\mathcal{G}}\right)$ as shown in Figs. 4 and 5, respectively. Here $\underline{\mathcal{G}}=\left(\underline{R}Y,\underline{S}Z\right)$, $\overline{\mathcal{G}}=\left(\overline{R}Y,\overline{S}Z\right)$, $\underline{\mathcal{H}}=\left(\underline{R}\widetilde{Y},\underline{S}\widetilde{Z}\right)$ and $\overline{\mathcal{H}}=\left(\overline{R}\widetilde{Y},\overline{S}\widetilde{Z}\right)$ in Figs. 4 and 5, depict IFRGs. The weight of the geodesic connecting the vertices of $\mathcal{G}=\left(\underline{\mathcal{G}},\overline{\mathcal{G}}\right)$ and $\mathcal{H}=\left(\underline{\mathcal{H}} ,\overline{\mathcal{H}}\right)$ are as follows, whose sum is minimal, according to direct computations:

$${ds}_{\underline{\mathcal{G}}}({p}_{1},{p}_{2})=(\text{0.5,0.2}), {ds}_{\underline{\mathcal{G}}}({p}_{2},{p}_{1})=(\text{1.0,0.3}),{ds}_{\overline{\mathcal{G}}}({p}_{1},{p}_{2})=(\text{0.7,0.0}), {ds}_{\overline{\mathcal{G}}}({p}_{2},{p}_{1})=(\text{1.2,0.0}),$$

$${ds}_{\underline{\mathcal{G}}}({p}_{1},{p}_{3})=(\text{1.0,0.4}), {ds}_{\underline{\mathcal{G}}}({p}_{3},{p}_{1})=(\text{0.5,0.2}),{ds}_{\overline{\mathcal{G}}}({p}_{1},{p}_{3})=(\text{1.4,0.0}), {ds}_{\overline{\mathcal{G}}}({p}_{3},{p}_{1})=(\text{1.2,0.0}),$$

$${ds}_{\underline{\mathcal{G}}}({p}_{1},{p}_{4})=(\text{1.0,0.3}), {ds}_{\underline{\mathcal{G}}}({p}_{4},{p}_{1})=(\text{0.5,0.2}),{ds}_{\overline{\mathcal{G}}}({p}_{1},{p}_{4})=(\text{1.2,0.0}), {ds}_{\overline{\mathcal{G}}}({p}_{4},{p}_{1})=(\text{0.7,0.0}),$$

$${ds}_{\underline{\mathcal{G}}}({p}_{2},{p}_{3})=(\text{0.5,0.2}), {ds}_{\underline{\mathcal{G}}}({p}_{3},{p}_{2})=(\text{1.0,0.4}),{ds}_{\overline{\mathcal{G}}}({p}_{2},{p}_{3})=(\text{0.7,0.0}), {ds}_{\overline{\mathcal{G}}}({p}_{3},{p}_{2})=(\text{1.9,0.0}),$$

$${ds}_{\underline{\mathcal{G}}}({p}_{2},{p}_{4})=(\text{0.5,0.1}), {ds}_{\underline{\mathcal{G}}}({p}_{4},{p}_{2})=(\text{1.0,0.4}),{ds}_{\overline{\mathcal{G}}}({p}_{2},{p}_{4})=(\text{0.5,0.0}), {ds}_{\overline{\mathcal{G}}}({p}_{4},{p}_{2})=(\text{1.4,0.0}),$$

$${ds}_{\underline{\mathcal{G}}}({p}_{3},{p}_{4})=(\text{0.5,0.1}), {ds}_{\underline{\mathcal{G}}}({p}_{4},{p}_{3})=(\text{1.5,0.6}),{ds}_{\overline{\mathcal{G}}}({p}_{3},{p}_{4})=(\text{0.5,0.0}), {ds}_{\overline{\mathcal{G}}}({p}_{4},{p}_{3})=(\text{2.1,0.0}).$$

and

$${ds}_{\underline{\mathcal{H}}}({p}_{1},{p}_{2})=(\text{0.5,0.2}), {ds}_{\underline{\mathcal{H}}}({p}_{2},{p}_{1})=(\text{1.0,0.4}),{ds}_{\overline{\mathcal{H}}}({p}_{1},{p}_{2})=(\text{0.7,0.0}), {ds}_{\overline{\mathcal{H}}}({p}_{2},{p}_{1})=(\text{1.1,0.0}),$$

$${ds}_{\underline{\mathcal{H}}}({p}_{1},{p}_{3})=(\text{1.0,0.4}), {ds}_{\underline{\mathcal{H}}}({p}_{3},{p}_{1})=(\text{0.5,0.2}),{ds}_{\overline{\mathcal{H}}}({p}_{1},{p}_{3})=(\text{1.4,0.0}), {ds}_{\overline{\mathcal{H}}}({p}_{3},{p}_{1})=(\text{0.4,0.0}),$$

$${ds}_{\underline{\mathcal{H}}}({p}_{1},{p}_{4})=(\text{1.0,0.3}), {ds}_{\underline{\mathcal{H}}}({p}_{4},{p}_{1})=(\text{0.0,0.0}),{ds}_{\overline{\mathcal{H}}}({p}_{1},{p}_{4})=(\text{1.2,0.0}), {ds}_{\overline{\mathcal{H}}}({p}_{4},{p}_{1})=(\text{0.0,0.0}),$$

$${ds}_{\underline{\mathcal{H}}}({p}_{2},{p}_{3})=(\text{0.5,0.2}), {ds}_{\underline{\mathcal{H}}}({p}_{3},{p}_{2})=(\text{1.0,0.4}),{ds}_{\overline{\mathcal{H}}}({p}_{2},{p}_{3})=(\text{0.7,0.0}),{ ds}_{\overline{\mathcal{H}}}({p}_{3},{p}_{2})=(\text{1.1,0.0}),$$

$${ds}_{\underline{\mathcal{H}}}({p}_{2},{p}_{4})=(\text{0.5,0.1}), {ds}_{\underline{\mathcal{H}}}({p}_{4},{p}_{2})=(\text{0.0,0.0}),{ds}_{\overline{\mathcal{H}}}({p}_{2},{p}_{4})=(\text{0.5,0.0}), {ds}_{\overline{\mathcal{H}}}({p}_{4},{p}_{2})=(\text{0.0,0.0}),$$

$${ds}_{\underline{\mathcal{H}}}({p}_{3},{p}_{4})=(\text{0.5,0.1}), {ds}_{\underline{\mathcal{H}}}({p}_{4},{p}_{3})=(\text{0.0,0.0}),{ds}_{\overline{\mathcal{H}}}({p}_{3},{p}_{4})=(\text{0.5,0.0}), {ds}_{\overline{\mathcal{H}}}({p}_{4},{p}_{3})=(\text{0.0,0.0}).$$

By above computations and from Figs. 4 and 5, we have

$$\mathcal{W}\mathcal{I}(\underline{\mathcal{G}})=3.5560, \mathcal{W}\mathcal{I}(\overline{\mathcal{G}})=7.5300, and \mathcal{W}\mathcal{I}(\mathcal{G})=11.0860.$$

$$\mathcal{W}\mathcal{I}(\underline{\mathcal{H}})=2.4320, \mathcal{W}\mathcal{I}(\overline{\mathcal{H}})=4.0850, and \mathcal{W}\mathcal{I}(\mathcal{H})=6.5170.$$

Clearly, from above calculations $\mathcal{W}\mathcal{I}(\mathcal{G})>\mathcal{W}\mathcal{I}(\mathcal{H})$.

### Theorem 3.4

Consider an IFRG ${\mathcal{G}}_{1}=\left(\underline{{\mathcal{G}}_{1}},\overline{{\mathcal{G}}_{1}}\right)$ and ${\mathcal{G}}_{2}=\left(\underline{{\mathcal{G}}_{2}},\overline{{\mathcal{G}}_{2}}\right)$ and ${\mathcal{G}}_{1}\cong {\mathcal{G}}_{2}$ then $\mathcal{W}\mathcal{I}({\mathcal{G}}_{1})=\mathcal{W}\mathcal{I}({\mathcal{G}}_{2})$.

### Proof

Let be two IFRGs ${\mathcal{G}}_{1}=\left(\underline{{\mathcal{G}}_{1}},\overline{{\mathcal{G}}_{1}}\right)$ and ${\mathcal{G}}_{2}=\left(\underline{{\mathcal{G}}_{2}},\overline{{\mathcal{G}}_{2}}\right)$. Also ${\mathcal{G}}_{1}=\left(\underline{{\mathcal{G}}_{1}},\overline{{\mathcal{G}}_{1}}\right)$ and ${\mathcal{G}}_{2}=\left(\underline{{\mathcal{G}}_{2}},\overline{{\mathcal{G}}_{2}}\right)$ are isomorphic graphs such that ${\mathcal{G}}_{1}\cong {\mathcal{G}}_{2}$. Then there is a pair of bijective mappings ($\underline{b}, \overline{b}) : P\to P$ such that.

$$\begin{aligned} ({\text{i}})\;\left( {\underline{R} Y_{1} } \right)^{M} (p_{1} ) = & \left( {\underline{R} Y_{2} } \right)^{M} (\underline{b} (p_{1} ))and\left( {\underline{R} Y_{1} } \right)^{N} (p_{1} ) = \left( {\underline{R} Y_{2} } \right)^{N} (\underline{b} (p_{1} )), \\ \left( {\underline{S} Z_{1} } \right)^{M} (p_{1} p_{2} ) = & \left( {\underline{S} Z_{2} } \right)^{M} (\underline{b} (p_{1} )\underline{b} (p_{2} ))and\left( {\underline{S} Z_{1} } \right)^{N} (p_{1} p_{2} ) = \left( {\underline{S} Z_{2} } \right)^{N} (\underline{b} (p_{1} )\underline{b} (p_{2} )),\forall p_{1} \in P,p_{1} p_{2} \in P \times P. \\ \end{aligned}$$

$$\begin{aligned} ({\text{ii}})\;\;\left( {\bar{R}Y_{1} } \right)^{M} (p_{1} ) = & \left( {\bar{R}Y_{2} } \right)^{M} (\bar{b}(p_{1} ))and\left( {\bar{R}Y_{1} } \right)^{N} (p_{1} ) = \left( {\bar{R}Y_{2} } \right)^{N} (\bar{b}(p_{1} )), \\ \left( {\bar{S}Z_{1} } \right)^{M} (p_{1} p_{2} ) = & \left( {\bar{S}Z_{2} } \right)^{M} (\bar{b}(p_{1} )\bar{b}(p_{2} ))and\left( {\bar{S}Z_{1} } \right)^{N} (p_{1} p_{2} ) = \left( {\bar{S}Z_{2} } \right)^{N} (\bar{b}(p_{1} )\bar{b}(p_{2} )),\forall p_{1} \in P,p_{1} p_{2} \in P \times P. \\ \end{aligned}$$

Firstly, we examine (i) using the notion of isomorphism provided above.

For ${p}_{1}$, ${p}_{2}\in {\left(\underline{R}{Y}_{1}\right)}^{M}, {\left(\underline{R}{Y}_{1}\right)}^{N}$ , let $\underline{{\text{C}}_{1}}$ be the directed ${p}_{1}-{p}_{2}$ path in $\underline{{\mathcal{G}}_{1}}$ which gives ${ds}_{\underline{{\text{M}\mathcal{G}}_{1}}}({p}_{1},{p}_{2}$), ${ds}_{\underline{{\text{N}\mathcal{G}}_{1}}}({p}_{1},{p}_{2}$).

Similarly for every directed edge ${\text{w}}_{1}{\text{w}}_{2}\in \underline{{\text{C}}_{1}}$ , there corresponds an directed edge $\underline{b}({\text{w}}_{1})\underline{b}({\text{w}}_{2})$ in $\underline{{\mathcal{G}}_{2}}$ such that ${\left(\underline{S}{Z}_{1}\right)}^{M}({\text{w}}_{1}{\text{w}}_{2}) = {\left(\underline{S}{Z}_{2}\right)}^{M}(\underline{b}({\text{w}}_{1})\underline{b}({\text{w}}_{2})) and {\left(\underline{S}{Z}_{1}\right)}^{N}({w}_{1}{w}_{2}) = {\left(\underline{S}{Z}_{2}\right)}^{N}(\underline{b}({\text{w}}_{1})\underline{b}({\text{w}}_{2})).$ Hence, it is easy to deduce that, in accordance with the ${p}_{1}-{p}_{2}$ path $\underline{{\text{C}}_{1}}$ in $\underline{{\mathcal{G}}_{1}}$, there exists a directed $\underline{b}({p}_{1})-\underline{b}({p}_{2})$ path $\underline{{\text{C}}_{2}}$ in $\underline{{\mathcal{G}}_{2}}$, such that the sum of the degree of membership of edges of $\underline{{\text{C}}_{2}}$ is minimal among all the shortest strong directed paths from $\underline{b}({\text{w}}_{1})\text{ to }\underline{b}({\text{w}}_{2})$.

Therefore, $${ds}_{\underline{{\text{M}\mathcal{G}}_{1}}}({p}_{1},{p}_{2}$$) =$${ds}_{\underline{{\text{M}\mathcal{G}}_{2}}}(\underline{b}({p}_{1})\underline{b}({p}_{2}))$$ and $${ds}_{\underline{{\text{N}\mathcal{G}}_{1}}}({p}_{1},{p}_{2}$$) = $${ds}_{\underline{{\text{N}\mathcal{G}}_{2}}}(\underline{b}({p}_{1})\underline{b}({p}_{2}))$$.

Hence

$$\mathcal{W}\mathcal{I}(\underline{{\mathcal{G}}_{1}})=\sum_{{p}_{1},{p}_{2}\in V(\underline{{\mathcal{G}}_{1}})}{\left(\underline{R}{Y}_{1}\right)}^{M}({p}_{1}){\left(\underline{R}{Y}_{1}\right)}^{M}({p}_{2}){ds}_{\underline{M{\mathcal{G}}_{1}}}({p}_{1},{p}_{2})+\sum_{{p}_{1},{p}_{2}\in V(\underline{{\mathcal{G}}_{1}})}{\left(\underline{R}{Y}_{1}\right)}^{N}({p}_{1}){\left(\underline{R}{Y}_{1}\right)}^{N}({p}_{2}){ds}_{\underline{N{\mathcal{G}}_{1}}}({p}_{1},{p}_{2}),$$

$$\begin{aligned} {\mathcal{W}\mathcal{I}}(\underline{{{\mathcal{G}}_{1} }} ) = & \sum\limits_{{\underline{b} (p_{1} ),\underline{b} (p_{2} ) \in V(\underline{{{\mathcal{G}}_{1} }} )}} {\left( {\underline{R} Y_{2} } \right)^{M} } \underline{b} (p_{1} )\left( {\underline{R} Y_{2} } \right)^{M} \underline{b} (p_{2} )ds_{{\underline{{M{\mathcal{G}}_{1} }} }} (\underline{b} (p_{1} ),\underline{b} (p_{2} )) \\ & \quad + \sum\limits_{{\underline{b} (p_{1} ),\underline{b} (p_{2} ) \in V(\underline{{{\mathcal{G}}_{1} }} )}} {\left( {\underline{R} Y_{2} } \right)^{N} } \underline{b} (p_{1} )\left( {\underline{R} Y_{2} } \right)^{N} \underline{b} (p_{2} )ds_{{\underline{{N{\mathcal{G}}_{1} }} }} (\underline{b} (p_{1} ),\underline{b} (p_{2} )) = {\mathcal{W}\mathcal{I}}(\underline{{{\mathcal{G}}_{2} }} ) \\ \end{aligned}$$

Thus, $\mathcal{W}\mathcal{I}(\underline{{\mathcal{G}}_{1}}) \cong \mathcal{W}\mathcal{I}(\underline{{\mathcal{G}}_{2}})$, Similarly, $\mathcal{W}\mathcal{I}(\overline{{\mathcal{G}}_{1}}) \cong \mathcal{W}\mathcal{I}(\overline{{\mathcal{G}}_{2}}).$ Hence, $\mathcal{W}\mathcal{I}({\mathcal{G}}_{1}) \cong \mathcal{W}\mathcal{I}({\mathcal{G}}_{2}).$

## Relationship between connectivity index and wiener index of an intuitionistic fuzzy rough graph

The relationship between the wiener index and connectivity index of an IFRG, $\mathcal{G}=\left(\underline{\mathcal{G}},\overline{\mathcal{G}}\right)$, is covered in this section. The next section contains some relevant findings and significant instances to help you understand how the wiener index and connectivity index are related. We see that the weiner index may be more, less, or equal to the connectivity index, depending on the properties of the network.

### Example 4.1

**Fig. 6:**
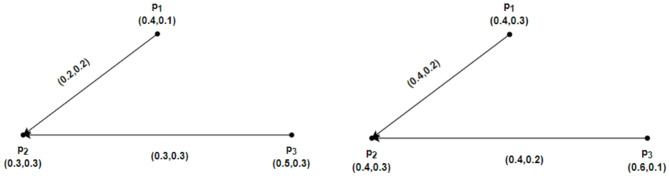
$$\mathcal{G}=\left(\underline{\mathcal{G}},\overline{\mathcal{G}}\right)$$ the IFRG.

Suppose $\mathcal{G}=\left(\underline{\mathcal{G}},\overline{\mathcal{G}}\right)$ be an IFRG on $u=\left\{{p}_{1}, {p}_{2},{p}_{3},{p}_{4}\right\}$. Therefore $\underline{\mathcal{G}}=\left(\underline{R}Y,\underline{S}Z\right)$ and $\overline{\mathcal{G}}=\left(\overline{R}Y,\overline{S}Z\right)$ in Fig. 6. depict IFRG. The $\mathcal{S}\mathcal{C}$ and the weight of the geodesic connecting the vertices of the IFRG whose total is minimal may be calculated directly as follows:

$${CONN}_{\underline{\mathcal{G}}({p}_{1},{p}_{2})}({p}_{1},{p}_{2})=(\text{0.2,0.2}), { CONN}_{\underline{\mathcal{G}}({p}_{2},{p}_{1})}({p}_{2},{p}_{1})=(\text{0.0,0.0}),$$

$${CONN}_{\overline{\mathcal{G}}({p}_{1},{p}_{2})}({p}_{1},{p}_{2})=(\text{0.4,0.2}), { CONN}_{\overline{\mathcal{G}}({p}_{2},{p}_{1})}({p}_{2},{p}_{1})=(\text{0.0,0.0}),$$

$${CONN}_{\underline{\mathcal{G}}({p}_{1},{p}_{3})}({p}_{1},{p}_{3})=(\text{0.0,0.0}), {CONN}_{\underline{\mathcal{G}}({p}_{3},{p}_{1})}({p}_{3},{p}_{1})=(\text{0.0,0.0}),$$

$${CONN}_{\overline{\mathcal{G}}({p}_{1},{p}_{3})}({p}_{1},{p}_{3})=(\text{0.0,0.0}), {CONN}_{\overline{\mathcal{G}}({p}_{3},{p}_{1})}({p}_{3},{p}_{1})=(\text{0.0,0.0}),$$

$${CONN}_{\underline{\mathcal{G}}({p}_{2},{p}_{3})}({p}_{2},{p}_{3})=(\text{0.0,0.0}), {CONN}_{\underline{\mathcal{G}}({p}_{3},{p}_{2})}({p}_{3},{p}_{2})=(\text{0.3,0.3}),$$

$${CONN}_{\overline{\mathcal{G}}({p}_{2},{p}_{3})}({p}_{2},{p}_{3})=(\text{0.0,0.0}), {CONN}_{\overline{\mathcal{G}}({p}_{3},{p}_{2})}({p}_{3},{p}_{2})=(\text{0.4,0.2}).$$

And

$${ds}_{\underline{\mathcal{G}}}\left({p}_{1},{p}_{2}\right)=\left(\text{0.2,0.2}\right), { ds}_{\underline{\mathcal{G}}}\left({p}_{2},{p}_{1}\right)=\left(\text{0.0,0.0}\right), {ds}_{\overline{\mathcal{G}}}\left({p}_{1},{p}_{2}\right)=\left(\text{0.4,0.2}\right),{ ds}_{\overline{\mathcal{G}}}({p}_{2},{p}_{1})=(\text{0.0,0.0}),$$

$${ds}_{\underline{\mathcal{G}}}\left({p}_{1},{p}_{3}\right)=\left(\text{0.0,0.0}\right), {ds}_{\underline{\mathcal{G}}}\left({p}_{3},{p}_{1}\right)=\left(\text{0.0,0.0}\right), {ds}_{\overline{\mathcal{G}}}({p}_{1},{p}_{3})=(\text{0.0,0.0}), {ds}_{\overline{\mathcal{G}}}({p}_{3},{p}_{1})=(\text{0.0,0.0}),$$

$${ds}_{\underline{\mathcal{G}}}\left({p}_{2},{p}_{3}\right)=\left(\text{0.0,0.0}\right), {ds}_{\underline{\mathcal{G}}}\left({p}_{3},{p}_{2}\right)=\left(\text{0.3,0.3}\right),{ds}_{\overline{\mathcal{G}}}({p}_{2},{p}_{3})=(\text{0.0,0.0}), {ds}_{\overline{\mathcal{G}}}({p}_{3},{p}_{2})=(\text{0.4,0.2}).$$

Form Fig. 6 and the calculations above yield

$$\mathcal{C}\mathcal{I}(\underline{\mathcal{G}})=0.1020, \mathcal{C}\mathcal{I}(\overline{\mathcal{G}})=0.1840, and \mathcal{C}\mathcal{I}(\mathcal{G})=0.2860.$$

$$\mathcal{W}\mathcal{I}(\underline{\mathcal{G}})=0.1020, \mathcal{W}\mathcal{I}(\overline{\mathcal{G}})=0.1840, and \mathcal{W}\mathcal{I}(\mathcal{G})=0.2860.$$

Thus, based on the computations above, $\mathcal{C}\mathcal{I}(\mathcal{G})=\mathcal{W}\mathcal{I}(\mathcal{G}).$

*Note*: In $\mathcal{G}=\left(\underline{\mathcal{G}},\overline{\mathcal{G}}\right)$ is an IFRG, if $\mathcal{C}\mathcal{I}(\underline{\mathcal{G}})=\mathcal{W}\mathcal{I}(\underline{\mathcal{G}})$ and $\mathcal{C}\mathcal{I}(\overline{\mathcal{G}})= \mathcal{W}\mathcal{I}(\overline{\mathcal{G}})$ then $\mathcal{C}\mathcal{I}(\mathcal{G})=\mathcal{W}\mathcal{I}(\mathcal{G}).$

It can be observed that $\mathcal{C}\mathcal{I}(\mathcal{G})=\mathcal{W}\mathcal{I}(\mathcal{G})$ for IFRG in Example 4.1. That being said, this equality need not always be the case. To illustrate this point, look at the example that follows.

### Example 4.2

**Fig. 7:**
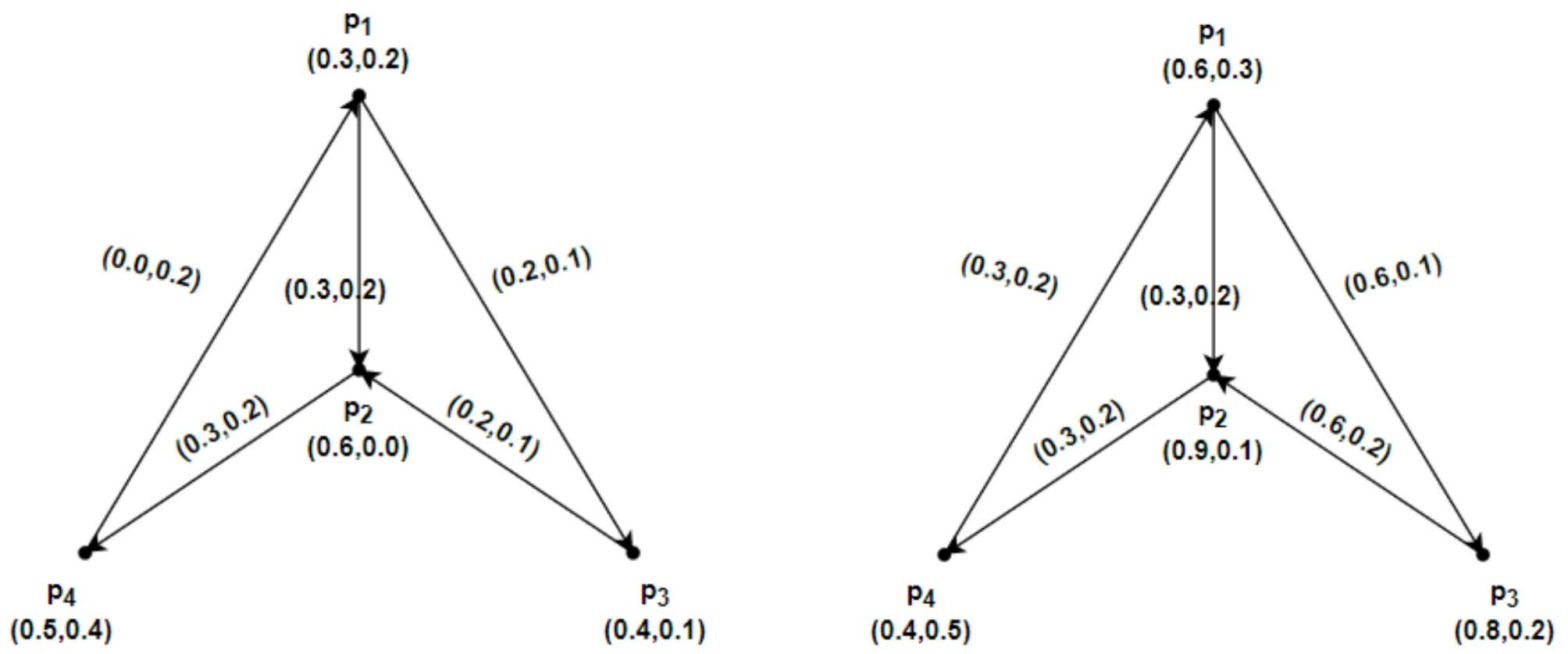
$\mathcal{G}=\left(\underline{\mathcal{G}},\overline{\mathcal{G}}\right)$ the IFRG with $\mathcal{C}\mathcal{I}(\underline{\mathcal{G}})<\mathcal{W}\mathcal{I}(\underline{G})$.

Suppose an IFRG $\mathcal{G}=\left(\underline{\mathcal{G}},\overline{\mathcal{G}}\right)$ on $u=\left\{{p}_{1}, {p}_{2},{p}_{3},{p}_{4}\right\}$ Therefore, $\underline{\mathcal{G}}=\left(\underline{R}Y,\underline{S}Z\right)$ and $\overline{\mathcal{G}}=\left(\overline{R}Y,\overline{S}Z\right)$ in Fig. 7. depict IFRGs. Show the IFRG. For the $\mathcal{S}\mathcal{C}$ and the weight of the geodesic linking the IFRG vertices whose total is minimal, direct computations get the following conclusions.

$${CONN}_{\underline{\mathcal{G}}({p}_{1},{p}_{2})}({p}_{1},{p}_{2})=(\text{0.3,0.1}), { CONN}_{\underline{\mathcal{G}}({p}_{2},{p}_{1})}({p}_{2},{p}_{1})=(\text{0.0,0.2}),$$

$${CONN}_{\overline{\mathcal{G}}({p}_{1},{p}_{2})}({p}_{1},{p}_{2})=(\text{0.6,0.2}), { CONN}_{\overline{\mathcal{G}}({p}_{2},{p}_{1})}({p}_{2},{p}_{1})=(\text{0.3,0.2}),$$

$${CONN}_{\underline{\mathcal{G}}({p}_{1},{p}_{3})}({p}_{1},{p}_{3})=(\text{0.2,0.1}), {CONN}_{\underline{\mathcal{G}}({p}_{3},{p}_{1})}({p}_{3},{p}_{1})=(\text{0.0,0.2}),$$

$${CONN}_{\overline{\mathcal{G}}({p}_{1},{p}_{3})}({p}_{1},{p}_{3})=(\text{0.6,0.1}), {CONN}_{\overline{\mathcal{G}}({p}_{3},{p}_{1})}({p}_{3},{p}_{1})=(\text{0.3,0.2}),$$

$${CONN}_{\underline{\mathcal{G}}({p}_{1},{p}_{4})}({p}_{1},{p}_{4})=(\text{0.3,0.2}), {CONN}_{\underline{\mathcal{G}}({p}_{4},{p}_{1})}({p}_{4},{p}_{1})=(\text{0.0,0.2}),$$

$${CONN}_{\overline{\mathcal{G}}({p}_{1},{p}_{4})}({p}_{1},{p}_{4})=(\text{0.3,0.2}), {CONN}_{\overline{\mathcal{G}}({p}_{4},{p}_{1})}({p}_{4},{p}_{1})=(\text{0.3,0.2}),$$

$${CONN}_{\underline{\mathcal{G}}({p}_{2},{p}_{3})}({p}_{2},{p}_{3})=(\text{0.0,0.2}), {CONN}_{\underline{\mathcal{G}}({p}_{3},{p}_{2})}({p}_{3},{p}_{2})=(\text{0.2,0.1}),$$

$${CONN}_{\overline{\mathcal{G}}({p}_{2},{p}_{3})}({p}_{2},{p}_{3})=(\text{0.3,0.2}), {CONN}_{\overline{\mathcal{G}}({p}_{3},{p}_{2})}({p}_{3},{p}_{2})=(\text{0.6,0.2}),$$

$${CONN}_{\underline{\mathcal{G}}({p}_{2},{p}_{4})}({p}_{2},{p}_{4})=(\text{0.3,0.2}), {CONN}_{\underline{\mathcal{G}}({p}_{4},{p}_{2})}({p}_{4},{p}_{2})=(\text{0.0,0.2}),$$

$${CONN}_{\overline{\mathcal{G}}({p}_{2},{p}_{4})}({p}_{2},{p}_{4})=(\text{0.3,0.2}), {CONN}_{\overline{\mathcal{G}}({p}_{4},{p}_{2})}({p}_{4},{p}_{2})=(\text{0.3,0.2}),$$

$${CONN}_{\underline{\mathcal{G}}({p}_{3},{p}_{4})}({p}_{3},{p}_{4})=(\text{0.2,0.2}), {CONN}_{\underline{\mathcal{G}}({p}_{4},{p}_{3})}({p}_{4},{p}_{3})=(\text{0.0,0.2}),$$

$${CONN}_{\overline{\mathcal{G}}({p}_{3},{p}_{4})}({p}_{3},{p}_{4})=(\text{0.3,0.2}), {CONN}_{\overline{\mathcal{G}}({p}_{4},{p}_{3})}({p}_{4},{p}_{3})=(\text{0.3,0.2}).$$

and

$${ds}_{\underline{\mathcal{G}}}({p}_{1},{p}_{2})=(\text{0.3,0.2}), {ds}_{\underline{\mathcal{G}}}({p}_{2},{p}_{1})=(\text{0.3,0.4}),{ds}_{\overline{\mathcal{G}}}({p}_{1},{p}_{2})=(\text{1.2,0.3}), {ds}_{\overline{\mathcal{G}}}({p}_{2},{p}_{1})=(\text{0.6,0.4}),$$

$${ds}_{\underline{\mathcal{G}}}({p}_{1},{p}_{3})=(\text{0.2,0.1}), {ds}_{\underline{\mathcal{G}}}({p}_{3},{p}_{1})=(\text{0.5,0.5}),{ds}_{\overline{\mathcal{G}}}({p}_{1},{p}_{3})=(\text{0.6,0.1}), {ds}_{\overline{\mathcal{G}}}({p}_{3},{p}_{1})=(\text{1.2,0.6}),$$

$${ds}_{\underline{\mathcal{G}}}({p}_{1},{p}_{4})=(\text{0.6,0.4}), {ds}_{\underline{\mathcal{G}}}({p}_{4},{p}_{1})=(\text{0.0,0.2}),{ds}_{\overline{\mathcal{G}}}({p}_{1},{p}_{4})=(\text{1.5,0.5}), {ds}_{\overline{\mathcal{G}}}({p}_{4},{p}_{1})=(\text{0.3,0.2}),$$

$${ds}_{\underline{\mathcal{G}}}({p}_{2},{p}_{3})=(\text{0.5,0.5}), {ds}_{\underline{\mathcal{G}}}({p}_{3},{p}_{2})=(\text{0.2,0.1}),{ds}_{\overline{\mathcal{G}}}({p}_{2},{p}_{3})=(\text{1.2,0.5}), {ds}_{\overline{\mathcal{G}}}({p}_{3},{p}_{2})=(\text{0.6,0.2}),$$

$${ds}_{\underline{\mathcal{G}}}({p}_{2},{p}_{4})=(\text{0.3,0.2}), {ds}_{\underline{\mathcal{G}}}({p}_{4},{p}_{2})=(\text{0.3,0.4}),{ds}_{\overline{\mathcal{G}}}({p}_{2},{p}_{4})=(\text{0.3,0.2}), {ds}_{\overline{\mathcal{G}}}({p}_{4},{p}_{2})=(\text{0.0,0.0}),$$

$${ds}_{\underline{\mathcal{G}}}({p}_{3},{p}_{4})=(\text{0.5,0.3}), {ds}_{\underline{\mathcal{G}}}({p}_{4},{p}_{3})=(\text{0.2,0.3}),{ds}_{\overline{\mathcal{G}}}({p}_{3},{p}_{4})=(\text{0.9,0.4}), {ds}_{\overline{\mathcal{G}}}({p}_{4},{p}_{3})=(\text{0.9,0.3}).$$

By above computations and from Fig. 7, we get

$$\mathcal{C}\mathcal{I}(\underline{\mathcal{G}})=0.2871, \mathcal{C}\mathcal{I}(\overline{\mathcal{G}})=2.2760, and \mathcal{C}\mathcal{I}(\mathcal{G})=2.5631.$$

$$\mathcal{W}\mathcal{I}(\underline{\mathcal{G}})=0.8540, \mathcal{W}\mathcal{I}(\overline{\mathcal{G}})=4.5100, and \mathcal{W}\mathcal{I}(\mathcal{G})=5.3640.$$

As seen from the computations above, $\mathcal{C}\mathcal{I}(\mathcal{G})<\mathcal{W}\mathcal{I}(\mathcal{G}).$

*Note*: It can be observed that $\mathcal{C}\mathcal{I}(\mathcal{G})<\mathcal{W}\mathcal{I}(\mathcal{G})$ for IFRG as illustrated in example.

This inequality, however, does not have to occur all of the time. Consider the following example to demonstrate this.

### Definition 4.3

If an IFRG $\mathcal{G}=\left(\underline{\mathcal{G}},\overline{\mathcal{G}}\right)$ is complete in $\underline{\mathcal{G}}$ and $\overline{\mathcal{G}}$, respectively, it is considered a complete intuitionistic fuzzy rough graph (CIFRG). In terms of math, we can write:

$${\left(\underline{S}Z\right)}^{M}\left({p}_{1}{p}_{2}\right)=min\left\{{\left(\underline{R}Y\right)}^{M}\left({p}_{1}\right),{\left(\underline{R}Y\right)}^{M}\left({p}_{2}\right)\right\},{\left(\underline{S}Z\right)}^{N}\left({p}_{1}{p}_{2}\right)=max\left\{{\left(\underline{R}Y\right)}^{N}\left({p}_{1}\right),{\left(\underline{R}Y\right)}^{N}\left({p}_{2}\right)\right\},$$

$${\left(\overline{S}Z\right)}^{M}\left({p}_{1}{p}_{2}\right)=min\left\{{\left(\overline{R}Y\right)}^{M}\left({p}_{1}\right),{\left(\overline{R}Y\right)}^{M}\left({p}_{2}\right)\right\},{\left(\overline{S}Z\right)}^{N}\left({p}_{1}{p}_{2}\right)=max\left\{{\left(\overline{R}Y\right)}^{N}\left({p}_{1}\right),{\left(\overline{R}Y\right)}^{N}\left({p}_{2}\right)\right\}.$$

### Definition 4.4

By calculating the average flow in this case, we may ascertain the degree of steady flow in the directed network. Thus, average wiener index, a new IFRG parameter, will be added.

Consider an IFRG $\mathcal{G}=\left(\underline{\mathcal{G}},\overline{\mathcal{G}}\right)$. The average wiener index $(\mathcal{A}\mathcal{W}\mathcal{I})$ of $\mathcal{G}=\left(\underline{\mathcal{G}},\overline{\mathcal{G}}\right)$ is

$$\mathcal{A}\mathcal{W}\mathcal{I}(\mathcal{G})=\mathcal{A}\mathcal{W}\mathcal{I}(\underline{\mathcal{G}})+\mathcal{A}\mathcal{W}\mathcal{I}(\overline{\mathcal{G}}),$$

where

$$\mathcal{A}\mathcal{W}\mathcal{I}(\underline{\mathcal{G}})=\frac{1}{\left(\genfrac{}{}{0pt}{}{n}{2}\right)}\sum_{{p}_{1},{p}_{2}\in V(\underline{\mathcal{G}})}(\underline{M})({p}_{1})(\underline{M})({p}_{2}){ds}_{\underline{M\mathcal{G}}}({p}_{1},{p}_{2})+\frac{1}{\left(\genfrac{}{}{0pt}{}{n}{2}\right)}\sum_{{p}_{1},{p}_{2}\in V(\underline{\mathcal{G}})}(\underline{N})({p}_{1})(\underline{N})({p}_{2}){ds}_{\underline{N\mathcal{G}}}({p}_{1},{p}_{2}),$$

$$\mathcal{A}\mathcal{W}\mathcal{I}(\overline{\mathcal{G}})=\frac{1}{\left(\genfrac{}{}{0pt}{}{n}{2}\right)}\sum_{{p}_{1},{p}_{2}\in V(\overline{\mathcal{G}})}(\overline{M})({p}_{1})(\overline{M})({p}_{2}){ds}_{\overline{M\mathcal{G}}}({p}_{1},{p}_{2})+\frac{1}{\left(\genfrac{}{}{0pt}{}{n}{2}\right)}\sum_{{p}_{1},{p}_{2}\in V(\overline{\mathcal{G}})}(\overline{N})({p}_{1})(\overline{N})({p}_{2}){ds}_{\overline{N\mathcal{G}}}({p}_{1},{p}_{2}).$$

### Example 4.5

Assume that $u=\left\{{p}_{1}, {p}_{2},{p}_{3},{p}_{4}\right\}$ has an IFRG $\mathcal{G}=\left(\underline{\mathcal{G}},\overline{\mathcal{G}}\right)$ as shown in Fig. 7. Therefore, $\underline{\mathcal{G}}=\left(\underline{R}Y,\underline{S}Z\right)$ and $\overline{\mathcal{G}}=\left(\overline{R}Y,\overline{S}Z\right)$ in Fig. 7. depict IFRGs. As a result, using direct computations and Fig. 7, we obtain.

$$\mathcal{W}\mathcal{I}(\underline{\mathcal{G}})=0.8540, \mathcal{W}\mathcal{I}(\overline{\mathcal{G}})=4.5100, and \mathcal{W}\mathcal{I}(\mathcal{G})=5.3640.$$

$$\mathcal{A}\mathcal{W}\mathcal{I}(\underline{\mathcal{G}})=0.1423, \mathcal{A}\mathcal{W}\mathcal{I}(\overline{\mathcal{G}})=0.7517, and \mathcal{A}\mathcal{W}\mathcal{I}(\mathcal{G})=0.8940.$$

*Note*: The average wiener index always has a value between 0 and 1.

### Example 4.6

**Fig. 8:**
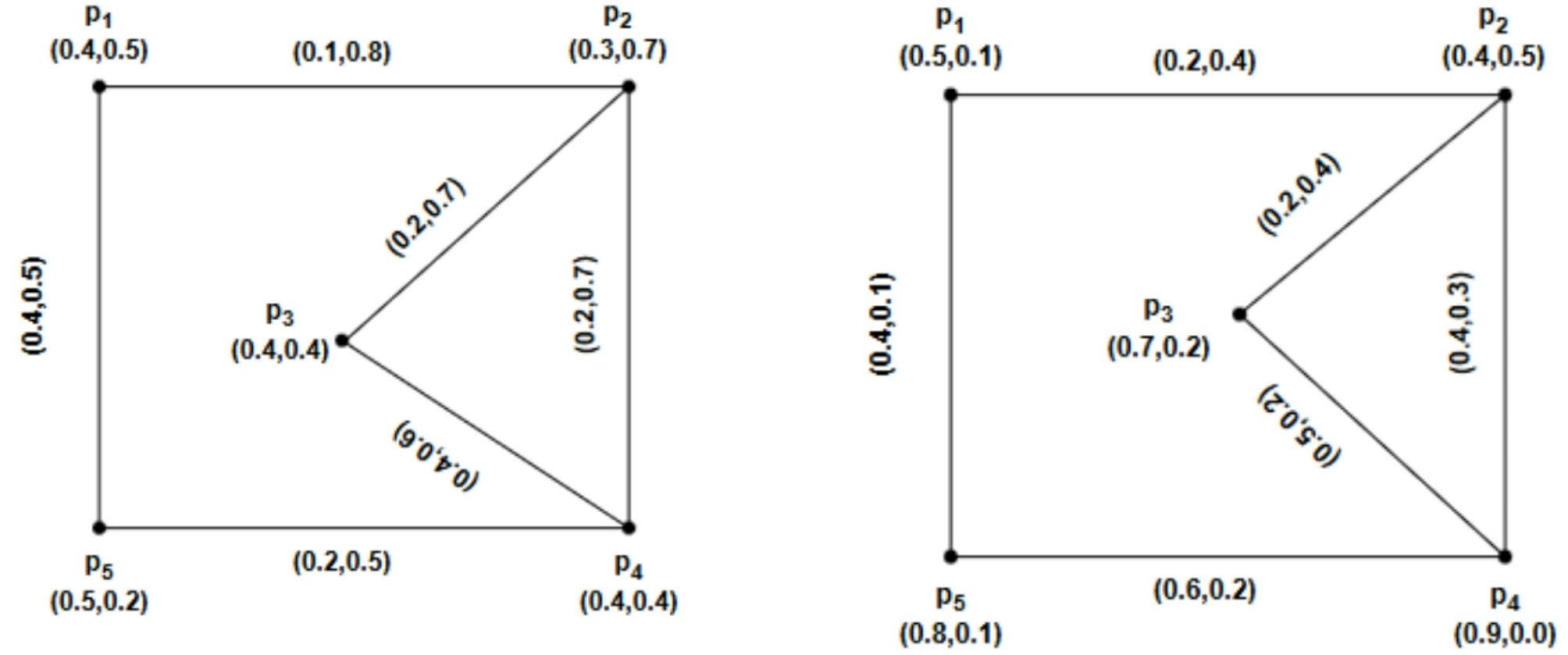
An IFRG with $\mathcal{W}\mathcal{I}(\mathcal{G})=8.4980$.

Using Fig. 8 we have prepared the following table after routine computations. From Table 1, we see that $\mathcal{W}\mathcal{I}(\mathcal{G})$ has been increased and decreased by deleting $\alpha -strong$ edges. We also have seen that $\mathcal{W}\mathcal{I}(\mathcal{G})$ is increased by removing $\beta -strong$ edges, but by deleting the $\delta -weak$ arc $\left({p}_{1},{p}_{2}\right)$, the strength of connectedness between every pair of vertices will remain the same, so that is the reason we have $\mathcal{W}\mathcal{I}(\mathcal{G})=\mathcal{W}\mathcal{I}(\mathcal{G}-\left({p}_{1},{p}_{2}\right))$.

**Table 1:** Values of Wiener index for $\mathcal{G}$ and $\mathcal{G}-\left({p}_{1},{p}_{2}\right)$.

Arcs	Types of arcs	Graphs	$\mathcal{W}\mathcal{I}(\underline{\mathcal{G}})$	$\mathcal{W}\mathcal{I}(\overline{\mathcal{G}})$	$\mathcal{W}\mathcal{I}(\mathcal{G})$
–	–	$\mathcal{G}$	2.788	5.710	8.4980
$\left({p}_{1},{p}_{2}\right)$	$\delta -weak$	$\mathcal{G}-\left({p}_{1},{p}_{2}\right)$	2.788	5.710	8.4980
$\left({p}_{2},{p}_{3}\right)$	$\beta -strong$	$\mathcal{G}-\left({p}_{2},{p}_{3}\right)$	3.015	7.241	10.2560
$\left({p}_{2},{p}_{4}\right)$	$\beta -strong$	$\mathcal{G}-\left({p}_{2},{p}_{4}\right)$	3.417	7.712	11.1290
$\left({p}_{3},{p}_{4}\right)$	$\alpha -strong$	$\mathcal{G}-\left({p}_{3},{p}_{4}\right)$	3.19	7.421	10.6110
$\left({p}_{4},{p}_{5}\right)$	$\alpha -strong$	$\mathcal{G}-\left({p}_{4},{p}_{5}\right)$	0.79	1.34	2.1300
$\left({p}_{1},{p}_{5}\right)$	$\alpha -strong$	$\mathcal{G}-\left({p}_{1},{p}_{5}\right)$	1.119	2.986	4.1050

## Some other indices of an intuitionistic fuzzy rough graph

### Definition 5.1

The membership degree is defined as the sum of the membership values of all the edges incident to the vertex. It is denoted as ${deg}_{\mathcal{G}}^{M}$. The non-membership degree is defined as the sum of the non-membership values of all the edges incident to the vertex. It is denoted as ${deg}_{\mathcal{G}}^{N}.$

### Definition 5.2

Let us consider an IFRG $\mathcal{G}=\left(\underline{\mathcal{G}},\overline{\mathcal{G}}\right)$. The T-hyper wiener index ($\mathcal{T}\mathcal{H}\mathcal{W}\mathcal{I})$ of $\mathcal{G}$ is defined as.

For lower approximation of IFRG:

$$\begin{aligned} {\mathcal{T}\mathcal{H}\mathcal{W}\mathcal{I}}(\underline{{\mathcal{G}}} ) = & \frac{1}{2}\left( {{\text{M}}{\mathcal{W}\mathcal{I}}(\underline{{\mathcal{G}}} ) + \sum\limits_{{p_{1} ,p_{2} \in V(\underline{{\mathcal{G}}} )}} {\left( {(\underline{M} )(p_{1} )(\underline{M} )(p_{2} )ds_{{\underline{{M{\mathcal{G}}}} }} (p_{1} ,p_{2} )} \right)^{2} } } \right) + \frac{1}{2}\left( {{\text{N}}{\mathcal{W}\mathcal{I}}(\underline{{\mathcal{G}}} ) + \sum\limits_{{p_{1} ,p_{2} \in V(\underline{{\mathcal{G}}} )}} {\left( {(\underline{N} )(p_{1} )(\underline{N} )(p_{2} )ds_{{\underline{{N{\mathcal{G}}}} }} (p_{1} ,p_{2} )} \right)^{2} } } \right) \\ = & \frac{1}{2}\left( {{\text{M}}{\mathcal{W}\mathcal{I}}(\underline{{\mathcal{G}}} ) + {\text{N}}{\mathcal{W}\mathcal{I}}(\underline{{\mathcal{G}}} ) + \sum\limits_{{p_{1} ,p_{2} \in V(\underline{{\mathcal{G}}} )}} {\left( {(\underline{M} )(p_{1} )(\underline{M} )(p_{2} )ds_{{\underline{{M{\mathcal{G}}}} }} (p_{1} ,p_{2} )} \right)^{2} } + \sum\limits_{{p_{1} ,p_{2} \in V(\underline{{\mathcal{G}}} )}} {\left( {(\underline{N} )(p_{1} )(\underline{N} )(p_{2} )ds_{{\underline{{N{\mathcal{G}}}} }} (p_{1} ,p_{2} )} \right)^{2} } } \right) \\ {\mathcal{T}\mathcal{H}\mathcal{W}\mathcal{I}}(\underline{{\mathcal{G}}} ) = & \frac{1}{2}\left( {{\mathcal{W}\mathcal{I}}(\underline{{\mathcal{G}}} ) + \sum\limits_{{p_{1} ,p_{2} \in V(\underline{{\mathcal{G}}} )}} {\left( {(\underline{M} )(p_{1} )(\underline{M} )(p_{2} )ds_{{\underline{{M{\mathcal{G}}}} }} (p_{1} ,p_{2} )} \right)^{2} } + \sum\limits_{{p_{1} ,p_{2} \in V(\underline{{\mathcal{G}}} )}} {\left( {(\underline{N} )(p_{1} )(\underline{N} )(p_{2} )ds_{{\underline{{N{\mathcal{G}}}} }} (p_{1} ,p_{2} )} \right)^{2} } } \right), \\ \end{aligned}$$

For upper approximation of IFRG:

$$\begin{aligned} {\mathcal{T}\mathcal{H}\mathcal{W}\mathcal{I}}(\overline{{\mathcal{G}}} ) = & \frac{1}{2}\left( {{\text{M}}{\mathcal{W}\mathcal{I}}(\overline{{\mathcal{G}}} ) + \sum\limits_{{p_{1} ,p_{2} \in V(\overline{{\mathcal{G}}} )}} {\left( {(\bar{M})(p_{1} )(\bar{M})(p_{2} )ds_{{\overline{{M{\mathcal{G}}}} }} (p_{1} ,p_{2} )} \right)^{2} } } \right) + \frac{1}{2}\left( {{\text{N}}{\mathcal{W}\mathcal{I}}(\overline{{\mathcal{G}}} ) + \sum\limits_{{p_{1} ,p_{2} \in V(\overline{{\mathcal{G}}} )}} {\left( {(\bar{N})(p_{1} )(\bar{N})(p_{2} )ds_{{\overline{{N{\mathcal{G}}}} }} (p_{1} ,p_{2} )} \right)^{2} } } \right) \\ = & \frac{1}{2}\left( {{\text{M}}{\mathcal{W}\mathcal{I}}(\overline{{\mathcal{G}}} ) + {\text{N}}{\mathcal{W}\mathcal{I}}(\overline{{\mathcal{G}}} ) + \sum\limits_{{p_{1} ,p_{2} \in V(\overline{{\mathcal{G}}} )}} {\left( {(\bar{M})(p_{1} )(\bar{M})(p_{2} )ds_{{\overline{{M{\mathcal{G}}}} }} (p_{1} ,p_{2} )} \right)^{2} } + \sum\limits_{{p_{1} ,p_{2} \in V(\overline{{\mathcal{G}}} )}} {\left( {(\bar{N})(p_{1} )(\bar{N})(p_{2} )ds_{{\overline{{N{\mathcal{G}}}} }} (p_{1} ,p_{2} )} \right)^{2} } } \right) \\ {\mathcal{T}\mathcal{H}\mathcal{W}\mathcal{I}}(\overline{{\mathcal{G}}} ) = & \frac{1}{2}\left( {{\mathcal{W}\mathcal{I}}(\overline{{\mathcal{G}}} ) + \sum\limits_{{p_{1} ,p_{2} \in V(\overline{{\mathcal{G}}} )}} {\left( {(\bar{M})(p_{1} )(\bar{M})(p_{2} )ds_{{\overline{{M{\mathcal{G}}}} }} (p_{1} ,p_{2} )} \right)^{2} } + \sum\limits_{{p_{1} ,p_{2} \in V(\overline{{\mathcal{G}}} )}} {\left( {(\bar{N})(p_{1} )(\bar{N})(p_{2} )ds_{{\overline{{N{\mathcal{G}}}} }} (p_{1} ,p_{2} )} \right)^{2} } } \right). \\ {\mathcal{T}\mathcal{H}\mathcal{W}\mathcal{I}}({\mathcal{G}}) = & {\mathcal{T}\mathcal{H}\mathcal{W}\mathcal{I}}(\underline{{\mathcal{G}}} ) + {\mathcal{T}\mathcal{H}\mathcal{W}\mathcal{I}}(\overline{{\mathcal{G}}} ), \\ \end{aligned}$$

### Definition 5.3

Let us consider an IFRG $\mathcal{G}=\left(\underline{\mathcal{G}},\overline{\mathcal{G}}\right)$. The Zegreb indices is defined as.

For lower approximation of IFRG:

$$\begin{aligned} {\mathcal{Z}\mathcal{I}}_{1} (\underline{{\mathcal{G}}} ) = & \sum\limits_{{p_{1} \in V(\underline{{\mathcal{G}}} )}} {\left( {(\underline{M} )(p_{1} )^{2} (\underline{N} )(p_{1} )^{2} } \right)} \left( {\left( {deg_{{\underline{{\mathcal{G}}} }}^{{\underline{M} }} (p_{1} )} \right)^{2} ,\left( {deg_{{\underline{{\mathcal{G}}} }}^{{\underline{N} }} (p_{1} )} \right)^{2} } \right) \\ = & \sum\limits_{{p_{1} \in V(\underline{{\mathcal{G}}} )}} {\left( {(\underline{M} )(p_{1} )^{2} \left( {deg_{{\underline{{\mathcal{G}}} }}^{{\underline{M} }} (p_{1} )} \right)^{2} + (\underline{N} )(p_{1} )^{2} \left( {deg_{{\underline{{\mathcal{G}}} }}^{{\underline{N} }} (p_{1} )} \right)^{2} } \right)} \\ {\mathcal{Z}\mathcal{I}}_{1} (\underline{{\mathcal{G}}} ) = & \sum\limits_{{p_{1} \in V(\underline{{\mathcal{G}}} )}} {\left( {(\underline{M} )(p_{1} )deg_{{\underline{{\mathcal{G}}} }}^{{\underline{M} }} (p_{1} )} \right)^{2} } + \sum\limits_{{p_{1} \in V(\underline{{\mathcal{G}}} )}} {\left( {(\underline{N} )(p_{1} )deg_{{\underline{{\mathcal{G}}} }}^{{\underline{N} }} (p_{1} )} \right)^{2} } \\ \end{aligned}$$

For upper approximation of IFRG:

$$\begin{aligned} {\mathcal{Z}\mathcal{I}}_{1} (\overline{{\mathcal{G}}} ) = & \sum\limits_{{p_{1} \in V(\overline{{\mathcal{G}}} )}} {\left( {(\bar{M})(p_{1} )^{2} (\bar{N})(p_{1} )^{2} } \right)} \left( {\left( {deg_{{\overline{{\mathcal{G}}} }}^{{\bar{M}}} (p_{1} )} \right)^{2} ,\left( {deg_{{\overline{{\mathcal{G}}} }}^{{\bar{N}}} (p_{1} )} \right)^{2} } \right) \\ = & \sum\limits_{{p_{1} \in V(\overline{{\mathcal{G}}} )}} {\left( {(\bar{M})(p_{1} )^{2} \left( {deg_{{\overline{{\mathcal{G}}} }}^{{\bar{M}}} (p_{1} )} \right)^{2} + (\bar{N})(p_{1} )^{2} \left( {deg_{{\overline{{\mathcal{G}}} }}^{{\bar{N}}} (p_{1} )} \right)^{2} } \right)} \\ {\mathcal{Z}\mathcal{I}}_{1} (\overline{{\mathcal{G}}} ) = & \sum\limits_{{p_{1} \in V(\overline{{\mathcal{G}}} )}} {\left( {(\bar{M})(p_{1} )deg_{{\overline{{\mathcal{G}}} }}^{{\bar{M}}} (p_{1} )} \right)^{2} } + \sum\limits_{{p_{1} \in V(\overline{{\mathcal{G}}} )}} {\left( {(\bar{N})(p_{1} )deg_{{\overline{{\mathcal{G}}} }}^{{\bar{N}}} (p_{1} )} \right)^{2} } \\ {\mathcal{Z}\mathcal{I}}_{1} ({\mathcal{G}}) = & {\mathcal{Z}\mathcal{I}}_{1} (\underline{{\mathcal{G}}} ) + {\mathcal{Z}\mathcal{I}}_{1} (\overline{{\mathcal{G}}} ), \\ \end{aligned}$$

For lower approximation of IFRG:

$$\begin{aligned} {\mathcal{Z}\mathcal{I}}_{2} (\underline{{\mathcal{G}}} ) = & \sum\limits_{{p_{1} ,p_{2} \in V(\underline{{\mathcal{G}}} )}} {\left( {\left( {(\underline{M} )(p_{1} )(\underline{N} )(p_{1} )} \right)\left( {(\underline{M} )(p_{2} )(\underline{N} )(p_{2} )} \right)\left( {deg_{{\underline{{\mathcal{G}}} }}^{{\underline{M} }} (p_{1} )deg_{{\underline{{\mathcal{G}}} }}^{{\underline{M} }} (p_{2} ),deg_{{\underline{{\mathcal{G}}} }}^{{\underline{N} }} (p_{1} )deg_{{\underline{{\mathcal{G}}} }}^{{\underline{N} }} (p_{2} )} \right)} \right)} \\ = & \sum\limits_{{p_{1} ,p_{2} \in V(\underline{{\mathcal{G}}} )}} {\left( {(\underline{M} )(p_{1} )(\underline{M} )(p_{2} )deg_{{\underline{{\mathcal{G}}} }}^{{\underline{M} }} (p_{1} )deg_{{\underline{{\mathcal{G}}} }}^{{\underline{M} }} (p_{2} ) + (\underline{N} )(p_{1} )(\underline{N} )(p_{2} )deg_{{\underline{{\mathcal{G}}} }}^{{\underline{N} }} (p_{1} )deg_{{\underline{{\mathcal{G}}} }}^{{\underline{N} }} (p_{2} )} \right)} \\ {\mathcal{Z}\mathcal{I}}_{2} (\underline{{\mathcal{G}}} ) = & \sum\limits_{{p_{1} ,p_{2} \in V(\underline{{\mathcal{G}}} )}} {\left( {(\underline{M} )(p_{1} )(\underline{M} )(p_{2} )deg_{{\underline{{\mathcal{G}}} }}^{{\underline{M} }} (p_{1} )deg_{{\underline{{\mathcal{G}}} }}^{{\underline{M} }} (p_{2} )} \right)} + \sum\limits_{{p_{1} ,p_{2} \in V(\underline{{\mathcal{G}}} )}} {\left( {(\underline{N} )(p_{1} )(\underline{N} )(p_{2} )deg_{{\underline{{\mathcal{G}}} }}^{{\underline{N} }} (p_{1} )deg_{{\underline{{\mathcal{G}}} }}^{{\underline{N} }} (p_{2} )} \right)} \\ \end{aligned}$$

For upper approximation of IFRG:

$$\begin{aligned} {\mathcal{Z}\mathcal{I}}_{2} (\overline{{\mathcal{G}}} ) = & \sum\limits_{{p_{1} ,p_{2} \in V(\overline{{\mathcal{G}}} )}} {\left( {\left( {(\bar{M})(p_{1} )(\bar{N})(p_{1} )} \right)\left( {(\bar{M})(p_{2} )(\bar{N})(p_{2} )} \right)\left( {deg_{{\overline{{\mathcal{G}}} }}^{{\bar{M}}} (p_{1} )deg_{{\overline{{\mathcal{G}}} }}^{{\bar{M}}} (p_{2} ),deg_{{\overline{{\mathcal{G}}} }}^{{\bar{N}}} (p_{1} )deg_{{\overline{{\mathcal{G}}} }}^{{\bar{N}}} (p_{2} )} \right)} \right)} \\ = & \sum\limits_{{p_{1} ,p_{2} \in V(\overline{{\mathcal{G}}} )}} {\left( {(\bar{M})(p_{1} )(\bar{M})(p_{2} )deg_{{\overline{{\mathcal{G}}} }}^{{\bar{M}}} (p_{1} )deg_{{\overline{{\mathcal{G}}} }}^{{\bar{M}}} (p_{2} ) + (\bar{N})(p_{1} )(\bar{N})(p_{2} )deg_{{\overline{{\mathcal{G}}} }}^{{\bar{N}}} (p_{1} )deg_{{\overline{{\mathcal{G}}} }}^{{\bar{N}}} (p_{2} )} \right)} \\ {\mathcal{Z}\mathcal{I}}_{2} (\overline{{\mathcal{G}}} ) = & \sum\limits_{{p_{1} ,p_{2} \in V(\overline{{\mathcal{G}}} )}} {\left( {(\bar{M})(p_{1} )(\bar{M})(p_{2} )deg_{{\overline{{\mathcal{G}}} }}^{{\bar{M}}} (p_{1} )deg_{{\overline{{\mathcal{G}}} }}^{{\bar{M}}} (p_{2} )} \right)} + \sum\limits_{{p_{1} ,p_{2} \in V(\overline{{\mathcal{G}}} )}} {\left( {(\bar{N})(p_{1} )(\bar{N})(p_{2} )deg_{{\overline{{\mathcal{G}}} }}^{{\bar{N}}} (p_{1} )deg_{{\overline{{\mathcal{G}}} }}^{{\bar{N}}} (p_{2} )} \right)} . \\ {\mathcal{Z}\mathcal{I}}_{2} ({\mathcal{G}}) = & {\mathcal{Z}\mathcal{I}}_{2} (\mathop {\mathcal{G}}\limits_{\_} ) + {\mathcal{Z}\mathcal{I}}_{2} (\overline{{\mathcal{G}}} ) \\ \end{aligned}$$

### Definition 5.4

Let us consider an IFRG $\mathcal{G}=\left(\underline{\mathcal{G}},\overline{\mathcal{G}}\right)$. The Randic index is given as.

For lower approximation of IFRG:

$$\begin{aligned} {\mathcal{R}\mathcal{I}}(\underline{{\mathcal{G}}} ) = & \sum\limits_{{p_{1} ,p_{2} \in V(\underline{{\mathcal{G}}} )}} {\left( {\left( {\underline{M} } \right)\left( {p_{1} } \right)^{{\frac{{ - 1}}{2}}} ,\left( {\underline{N} } \right)\left( {p_{1} } \right)^{{\frac{{ - 1}}{2}}} } \right)} \left( {\left( {\underline{M} } \right)\left( {p_{2} } \right)^{{\frac{{ - 1}}{2}}} ,\left( {\underline{N} } \right)\left( {p_{2} } \right)^{{\frac{{ - 1}}{2}}} } \right) \times \left( {\left( {deg_{{\underline{{\mathcal{G}}} }}^{{\underline{M} }} (p_{1} )deg_{{\underline{{\mathcal{G}}} }}^{{\underline{M} }} (p_{2} )} \right)^{{\frac{{ - 1}}{2}}} \left( {deg_{{\underline{{\mathcal{G}}} }}^{{\underline{N} }} (p_{1} )deg_{{\underline{{\mathcal{G}}} }}^{{\underline{N} }} (p_{2} )} \right)^{{\frac{{ - 1}}{2}}} } \right) \\ = & \sum\limits_{{p_{1} ,p_{2} \in V(\underline{{\mathcal{G}}} )}} {\left( {\left( {\underline{M} } \right)\left( {p_{1} } \right)^{{\frac{{ - 1}}{2}}} ,\left( {\underline{M} } \right)\left( {p_{2} } \right)^{{\frac{{ - 1}}{2}}} \left( {deg_{{\underline{{\mathcal{G}}} }}^{{\underline{M} }} (p_{1} )deg_{{\underline{{\mathcal{G}}} }}^{{\underline{M} }} (p_{2} )} \right)^{{\frac{{ - 1}}{2}}} + \left( {\underline{N} } \right)\left( {p_{1} } \right)^{{\frac{{ - 1}}{2}}} ,\left( {\underline{N} } \right)\left( {p_{2} } \right)^{{\frac{{ - 1}}{2}}} \left( {deg_{{\underline{{\mathcal{G}}} }}^{{\underline{N} }} (p_{1} )deg_{{\underline{{\mathcal{G}}} }}^{{\underline{N} }} (p_{2} )} \right)^{{\frac{{ - 1}}{2}}} } \right)} \\ = & \sum\limits_{{p_{1} ,p_{2} \in V(\underline{{\mathcal{G}}} )}} {\left( {\left( {\underline{{\text{M}}} } \right)\left( {{\text{p}}_{1} } \right)^{{\frac{{ - 1}}{2}}} ,\left( {\underline{{\text{M}}} } \right)\left( {{\text{p}}_{2} } \right)^{{\frac{{ - 1}}{2}}} \left( {deg_{{\underline{{\mathcal{G}}} }}^{{\underline{M} }} (p_{1} )deg_{{\underline{{\mathcal{G}}} }}^{{\underline{M} }} (p_{2} )} \right)^{{\frac{{ - 1}}{2}}} } \right)} + \sum\limits_{{p_{1} ,p_{2} \in V(\underline{{\mathcal{G}}} )}} {\left( {\left( {\underline{N} } \right)\left( {p_{1} } \right)^{{\frac{{ - 1}}{2}}} ,\left( {\underline{N} } \right)\left( {p_{2} } \right)^{{\frac{{ - 1}}{2}}} \left( {deg_{{\underline{{\mathcal{G}}} }}^{{\underline{N} }} (p_{1} )deg_{{\underline{{\mathcal{G}}} }}^{{\underline{N} }} (p_{2} )} \right)^{{\frac{{ - 1}}{2}}} } \right)} \\ {\mathcal{R}\mathcal{I}}(\underline{{\mathcal{G}}} ) = & \sum\limits_{{p_{1} ,p_{2} \in V(\underline{{\mathcal{G}}} )}} {\left( {\frac{1}{{\sqrt {\left( {(\underline{M} )(p_{1} )(\underline{M} )(p_{2} )deg_{{\underline{{\mathcal{G}}} }}^{{\underline{M} }} (p_{1} )deg_{{\underline{{\mathcal{G}}} }}^{{\underline{M} }} (p_{2} )} \right)} }}} \right)} + \sum\limits_{{p_{1} ,p_{2} \in V(\underline{{\mathcal{G}}} )}} {\left( {\frac{1}{{\sqrt {\left( {(\underline{N} )(p_{1} )(\underline{N} )(p_{2} )deg_{{\underline{{\mathcal{G}}} }}^{{\underline{N} }} (p_{1} )deg_{{\underline{{\mathcal{G}}} }}^{{\underline{N} }} (p_{2} )} \right)} }}} \right)} \\ \end{aligned}$$

For upper approximation of IFRG:

$$\begin{aligned} {\mathcal{R}\mathcal{I}}(\overline{{\mathcal{G}}} ) = & \sum\limits_{{p_{1} ,p_{2} \in V(\overline{{\mathcal{G}}} )}} {\left( {(\bar{M})\left( {p_{1} } \right)^{{\frac{{ - 1}}{2}}} ,(\bar{N})\left( {p_{1} } \right)^{{\frac{{ - 1}}{2}}} } \right)} \left( {(\bar{M})\left( {p_{2} } \right)^{{\frac{{ - 1}}{2}}} ,(\bar{N})\left( {p_{2} } \right)^{{\frac{{ - 1}}{2}}} } \right) \times \left( {\left( {deg_{{\overline{{\mathcal{G}}} }}^{{\bar{M}}} (p_{1} )deg_{{\overline{{\mathcal{G}}} }}^{{\bar{M}}} (p_{2} )} \right)^{{\frac{{ - 1}}{2}}} \left( {deg_{{\overline{{\mathcal{G}}} }}^{{\bar{N}}} (p_{1} )deg_{{\overline{{\mathcal{G}}} }}^{{\bar{N}}} (p_{2} )} \right)^{{\frac{{ - 1}}{2}}} } \right) \\ = & \sum\limits_{{p_{1} ,p_{2} \in V(\overline{{\mathcal{G}}} )}} {\left( {(\bar{M})\left( {p_{1} } \right)^{{\frac{{ - 1}}{2}}} ,(\bar{M})\left( {p_{2} } \right)^{{\frac{{ - 1}}{2}}} \left( {deg_{{\overline{{\mathcal{G}}} }}^{{\bar{M}}} (p_{1} )deg_{{\overline{{\mathcal{G}}} }}^{{\bar{M}}} (p_{2} )} \right)^{{\frac{{ - 1}}{2}}} } \right.} \\ & \quad + \left. {(\bar{N})\left( {p_{1} } \right)^{{\frac{{ - 1}}{2}}} ,(\bar{N})\left( {p_{2} } \right)^{{\frac{{ - 1}}{2}}} \left( {deg_{{\overline{{\mathcal{G}}} }}^{{\bar{N}}} (p_{1} )deg_{{\overline{{\mathcal{G}}} }}^{{\bar{N}}} (p_{2} )} \right)^{{\frac{{ - 1}}{2}}} } \right) \\ = & \sum\limits_{{p_{1} ,p_{2} \in V(\overline{{\mathcal{G}}} )}} {\left( {(\bar{M})\left( {{\text{p}}_{1} } \right)^{{\frac{{ - 1}}{2}}} ,(\bar{M})\left( {{\text{p}}_{2} } \right)^{{\frac{{ - 1}}{2}}} \left( {deg_{{\overline{{\mathcal{G}}} }}^{{\bar{M}}} (p_{1} )deg_{{\overline{{\mathcal{G}}} }}^{{\bar{M}}} (p_{2} )} \right)^{{\frac{{ - 1}}{2}}} } \right)} \\ & \quad + \sum\limits_{{p_{1} ,p_{2} \in V(\overline{{\mathcal{G}}} )}} {\left( {(\bar{N})\left( {p_{1} } \right)^{{\frac{{ - 1}}{2}}} ,(\bar{N})\left( {p_{2} } \right)^{{\frac{{ - 1}}{2}}} \left( {deg_{{\overline{{\mathcal{G}}} }}^{{\bar{N}}} (p_{1} )deg_{{\overline{{\mathcal{G}}} }}^{{\bar{N}}} (p_{2} )} \right)^{{\frac{{ - 1}}{2}}} } \right)} \\ \end{aligned}$$

$$\begin{aligned} {\mathcal{R}\mathcal{I}}(\overline{{\mathcal{G}}} ) = & \sum\limits_{{p_{1} ,p_{2} \in V(\overline{{\mathcal{G}}} )}} {\left( {\frac{1}{{\sqrt {\left( {(\bar{M})(p_{1} )(\bar{M})(p_{2} )deg_{{\overline{{\mathcal{G}}} }}^{{\bar{M}}} (p_{1} )deg_{{\overline{{\mathcal{G}}} }}^{{\bar{M}}} (p_{2} )} \right)} }}} \right)} + \sum\limits_{{p_{1} ,p_{2} \in V(\overline{{\mathcal{G}}} )}} {\left( {\frac{1}{{\sqrt {\left( {(\bar{N})(p_{1} )(\bar{N})(p_{2} )deg_{{\overline{{\mathcal{G}}} }}^{{\bar{N}}} (p_{1} )deg_{{\overline{{\mathcal{G}}} }}^{{\bar{N}}} (p_{2} )} \right)} }}} \right)} \\ {\mathcal{R}\mathcal{I}}({\mathcal{G}}) = & {\mathcal{R}\mathcal{I}}({\underline{\mathcal{G}} }) + {\mathcal{R}\mathcal{I}}(\overline{{\mathcal{G}}} ), \\ \end{aligned}$$

### Definition 5.5

Let us consider an IFRG $\mathcal{G}=\left(\underline{\mathcal{G}},\overline{\mathcal{G}}\right)$. The Harmonic index is given as.

For lower approximation of IFRG:

$$\begin{aligned} {\mathcal{H}\mathcal{I}}(\underline{{\mathcal{G}}} ) = & \sum\limits_{{p_{1} ,p_{2} \in V(\underline{{\mathcal{G}}} )}} {\left( {\frac{1}{{(\underline{M} )(p_{1} )}}.\frac{1}{{(\underline{N} )(p_{1} )}}.\frac{1}{{(\underline{M} )(p_{2} )}}.\frac{1}{{(\underline{N} )(p_{2} )}}} \right)} \times \left( {\frac{2}{{(deg_{{\underline{{\mathcal{G}}} }}^{{\underline{M} }} (p_{1} ) + deg_{{\underline{{\mathcal{G}}} }}^{{\underline{M} }} (p_{2} ))}}.\frac{2}{{(deg_{{\underline{{\mathcal{G}}} }}^{{\underline{N} }} (p_{1} ) + deg_{{\underline{{\mathcal{G}}} }}^{{\underline{N} }} (p_{2} ))}}} \right) \\ = & \sum\limits_{{p_{1} ,p_{2} \in V(\underline{{\mathcal{G}}} )}} {\left( {\frac{1}{{(\underline{M} )(p_{1} )}}.\frac{1}{{(\underline{M} )(p_{2} )}}.\frac{2}{{(deg_{{\underline{{\mathcal{G}}} }}^{{\underline{M} }} (p_{1} ) + deg_{{\underline{{\mathcal{G}}} }}^{{\underline{M} }} (p_{2} ))}} + \frac{1}{{(\underline{N} )(p_{1} )}}.\frac{1}{{(\underline{N} )(p_{2} )}}.\frac{2}{{(deg_{{\underline{{\mathcal{G}}} }}^{{\underline{N} }} (p_{1} ) + deg_{{\underline{{\mathcal{G}}} }}^{{\underline{N} }} (p_{2} ))}}} \right)} \\ = & \sum\limits_{{p_{1} ,p_{2} \in V(\underline{{\mathcal{G}}} )}} {\left( {\frac{1}{{(\underline{M} )(p_{1} )}}.\frac{1}{{(\underline{M} )(p_{2} )}}.\frac{2}{{(deg_{{\underline{{\mathcal{G}}} }}^{{\underline{M} }} (p_{1} ) + deg_{{\underline{{\mathcal{G}}} }}^{{\underline{M} }} (p_{2} ))}}} \right)} + \sum\limits_{{p_{1} ,p_{2} \in V(\underline{{\mathcal{G}}} )}} {\left( {\frac{1}{{(\underline{N} )(p_{1} )}}.\frac{1}{{(\underline{N} )(p_{2} )}}.\frac{2}{{(deg_{{\underline{{\mathcal{G}}} }}^{{\underline{N} }} (p_{1} ) + deg_{{\underline{{\mathcal{G}}} }}^{{\underline{N} }} (p_{2} ))}}} \right)} \\ {\mathcal{H}\mathcal{I}}(\underline{{\mathcal{G}}} ) = & \sum\limits_{{p_{1} ,p_{2} \in V(\underline{{\mathcal{G}}} )}} {\left( {\frac{2}{{(\underline{M} )(p_{1} )(\underline{M} )(p_{2} )(deg_{{\underline{{\mathcal{G}}} }}^{{\underline{M} }} (p_{1} ) + deg_{{\underline{{\mathcal{G}}} }}^{{\underline{M} }} (p_{2} ))}}} \right)} + \sum\limits_{{p_{1} ,p_{2} \in V(\underline{{\mathcal{G}}} )}} {\left( {\frac{2}{{(\underline{N} )(p_{1} )(\underline{N} )(p_{2} )(deg_{{\underline{{\mathcal{G}}} }}^{{\underline{N} }} (p_{1} ) + deg_{{\underline{{\mathcal{G}}} }}^{{\underline{N} }} (p_{2} ))}}} \right)} \\ \end{aligned}$$

For upper approximation of IFRG:

$$\begin{aligned} {\mathcal{H}\mathcal{I}}(\overline{{\mathcal{G}}} ) = & \sum\limits_{{p_{1} ,p_{2} \in V(\overline{{\mathcal{G}}} )}} {\left( {\frac{1}{{(\bar{M})(p_{1} )}}.\frac{1}{{(\bar{N})(p_{1} )}}.\frac{1}{{(\bar{M})(p_{2} )}}.\frac{1}{{(\bar{N})(p_{2} )}}} \right)} \times \left( {\frac{2}{{(deg_{{\underline{{\mathcal{G}}} }}^{{\bar{M}}} (p_{1} ) + deg_{{\underline{{\mathcal{G}}} }}^{{\bar{M}}} (p_{2} ))}}.\frac{2}{{(deg_{{\overline{{\mathcal{G}}} }}^{{\bar{N}}} (p_{1} ) + deg_{{\overline{{\mathcal{G}}} }}^{{\bar{N}}} (p_{2} ))}}} \right) \\ = & \sum\limits_{{p_{1} ,p_{2} \in V(\overline{{\mathcal{G}}} )}} {\left( {\frac{1}{{(\bar{M})(p_{1} )}}.\frac{1}{{(\bar{M})(p_{2} )}}.\frac{2}{{(deg_{{\overline{{\mathcal{G}}} }}^{{\bar{M}}} (p_{1} ) + deg_{{\overline{{\mathcal{G}}} }}^{{\bar{M}}} (p_{2} ))}} + \frac{1}{{(\bar{N})(p_{1} )}}.\frac{1}{{(\bar{N})(p_{2} )}}.\frac{2}{{(deg_{{\overline{{\mathcal{G}}} }}^{{\bar{N}}} (p_{1} ) + deg_{{\overline{{\mathcal{G}}} }}^{{\bar{N}}} (p_{2} ))}}} \right)} \\ = & \sum\limits_{{p_{1} ,p_{2} \in V(\overline{{\mathcal{G}}} )}} {\left( {\frac{1}{{(\bar{M})(p_{1} )}}.\frac{1}{{(\bar{M})(p_{2} )}}.\frac{2}{{(deg_{{\overline{{\mathcal{G}}} }}^{{\bar{M}}} (p_{1} ) + deg_{{\overline{{\mathcal{G}}} }}^{{\bar{M}}} (p_{2} ))}}} \right)} + \sum\limits_{{p_{1} ,p_{2} \in V(\overline{{\mathcal{G}}} )}} {\left( {\frac{1}{{(\bar{N})(p_{1} )}}.\frac{1}{{(\bar{N})(p_{2} )}}.\frac{2}{{(deg_{{\overline{{\mathcal{G}}} }}^{{\bar{N}}} (p_{1} ) + deg_{{\overline{{\mathcal{G}}} }}^{{\bar{N}}} (p_{2} ))}}} \right)} \\ {\mathcal{H}\mathcal{I}}(\overline{{\mathcal{G}}} ) = & \sum\limits_{{p_{1} ,p_{2} \in V(\overline{{\mathcal{G}}} )}} {\left( {\frac{2}{{(\bar{M})(p_{1} )(\bar{M})(p_{2} )(deg_{{\overline{{\mathcal{G}}} }}^{{\bar{M}}} (p_{1} ) + deg_{{\overline{{\mathcal{G}}} }}^{{\bar{M}}} (p_{2} ))}}} \right)} + \sum\limits_{{p_{1} ,p_{2} \in V(\overline{{\mathcal{G}}} )}} {\left( {\frac{2}{{(\bar{N})(p_{1} )(\bar{N})(p_{2} )(deg_{{\overline{{\mathcal{G}}} }}^{{\bar{N}}} (p_{1} ) + deg_{{\overline{{\mathcal{G}}} }}^{{\bar{N}}} (p_{2} ))}}} \right)} . \\ {\mathcal{H}\mathcal{I}}({\mathcal{G}}) = & {\mathcal{H}\mathcal{I}}(\underline{{\mathcal{G}}} ) + {\mathcal{H}\mathcal{I}}(\overline{{\mathcal{G}}} ) \\ \end{aligned}$$

### Example 5.6

**Fig. 9:**
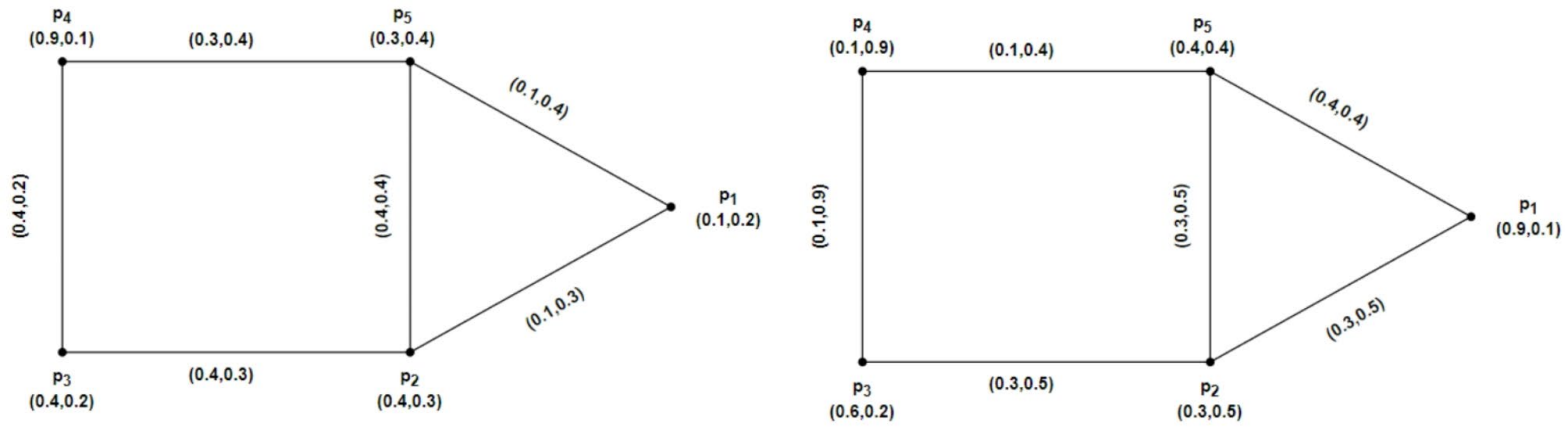
$$\mathcal{G}=\left(\underline{\mathcal{G}},\overline{\mathcal{G}}\right)$$ the IFRG.

For the IFRG in Fig. 9, $\mathcal{W}\mathcal{I}(\mathcal{G})=6.4540$, $\mathcal{T}\mathcal{H}\mathcal{W}\mathcal{I}(\mathcal{G})= 3.8073, {\mathcal{Z}\mathcal{I}}_{1}(\mathcal{G})= 3.8310,$ ${\mathcal{Z}\mathcal{I}}_{2}(\mathcal{G})= 9.5428,$ $\mathcal{R}\mathcal{I}(\mathcal{G})= 234.9689,$ $\mathcal{H}\mathcal{I}(\mathcal{G})= 958.1543.$

## Removal of vertices from an intuitionistic fuzzy rough graph and its impact on the wiener index and average wiener index

Although it truly depends on the kind of vertices we are removing, we may assume that the removal of vertices will have an impact on the wiener index.

### Example 6.1

**Fig. 10:**
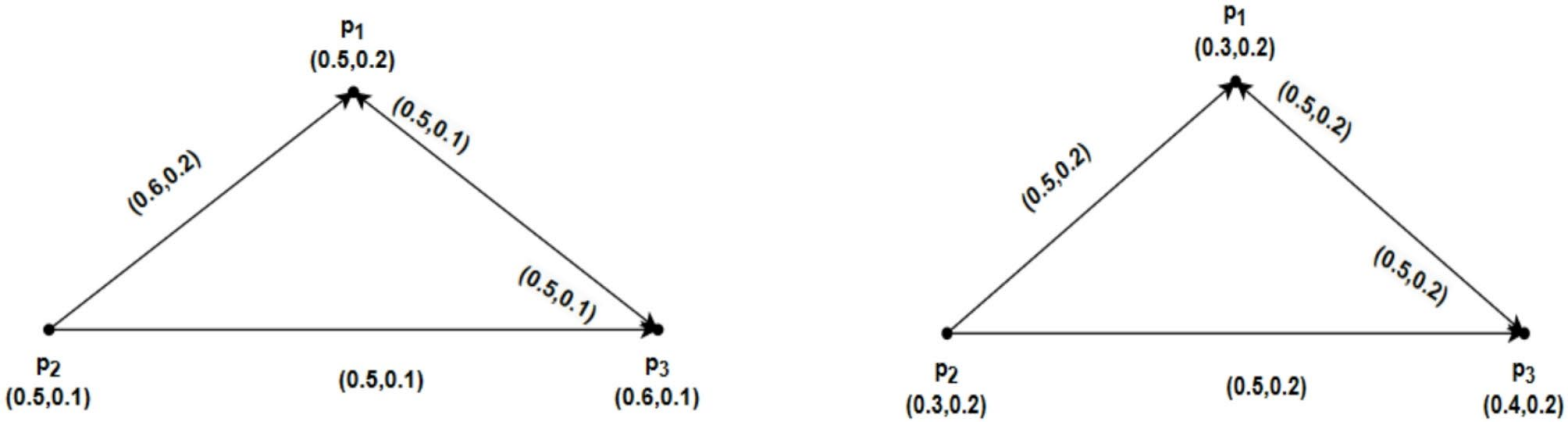
$$\mathcal{G}=\left(\underline{\mathcal{G}},\overline{\mathcal{G}}\right)$$ the IFRG.

As illustrated in Fig. 10. consider the IFRG $\mathcal{G}=\left(\underline{\mathcal{G}},\overline{\mathcal{G}}\right).$ The weight of the geodesic connecting the vertices of $\mathcal{G}$ whose total is smallest may be calculated directly as follows:

$${ds}_{\underline{\mathcal{G}}}({p}_{1},{p}_{2})=(\text{0.0,0.0}), {ds}_{\underline{\mathcal{G}}}({p}_{2},{p}_{1})=(\text{0.5,0.2}),{ds}_{\overline{\mathcal{G}}}({p}_{1},{p}_{2})=(\text{0.0,0.0}), {ds}_{\overline{\mathcal{G}}}({p}_{2},{p}_{1})=(\text{0.5,0.2}),$$

$${ds}_{\underline{\mathcal{G}}}({p}_{1},{p}_{3})=(\text{0.5,0.1}), {ds}_{\underline{\mathcal{G}}}({p}_{3},{p}_{1})=(\text{0.5,0.1}),{ds}_{\overline{\mathcal{G}}}({p}_{1},{p}_{3})=(\text{0.5,0.2}), {ds}_{\overline{\mathcal{G}}}({p}_{3},{p}_{1})=(\text{0.5,0.2}),$$

$${ds}_{\underline{\mathcal{G}}}({p}_{2},{p}_{3})=(\text{0.5,0.1}), {ds}_{\underline{\mathcal{G}}}({p}_{3},{p}_{2})=(\text{0.0,0.0}),{ds}_{\overline{\mathcal{G}}}({p}_{2},{p}_{3})=(\text{0.5,0.2}), {ds}_{\overline{\mathcal{G}}}({p}_{3},{p}_{2})=(\text{0.0,0.0}).$$

Based on the calculations above and Fig. 10, we have

$$\mathcal{W}\mathcal{I}(\underline{\mathcal{G}})=0.6070, \mathcal{W}\mathcal{I}(\overline{\mathcal{G}})=0.2570, \mathcal{W}\mathcal{I}(\mathcal{G})=0.8640.$$

$$\mathcal{A}\mathcal{W}\mathcal{I}(\underline{\mathcal{G}})=0.2023, \mathcal{A}\mathcal{W}\mathcal{I}(\overline{\mathcal{G}})=0.0857, \mathcal{A}\mathcal{W}\mathcal{I}(\mathcal{G})=0.2880.$$

As we remove the vertex ${p}_{1}$ from a graph, the nature of the remaining arcs also changed.

$${ds}_{\underline{\mathcal{G}}}({p}_{2},{p}_{3})=(\text{0.5,0.1}), {ds}_{\underline{\mathcal{G}}}({p}_{3},{p}_{2})=(\text{0.0,0.0}),{ds}_{\overline{\mathcal{G}}}({p}_{2},{p}_{3})=(\text{0.5,0.2}), {ds}_{\overline{\mathcal{G}}}({p}_{3},{p}_{2})=(\text{0.0,0.0}).$$

$$\mathcal{W}\mathcal{I}(\underline{\mathcal{G}}-{p}_{1})=0.1947, \mathcal{W}\mathcal{I}(\overline{\mathcal{G}}-{p}_{1})=0.0857, \mathcal{W}\mathcal{I}(\mathcal{G}-{p}_{1})=0.2804.$$

$$\mathcal{A}\mathcal{W}\mathcal{I}(\underline{\mathcal{G}}-{p}_{1})=0.1947, \mathcal{A}\mathcal{W}\mathcal{I}(\overline{\mathcal{G}}-{p}_{1})=0.0857, \mathcal{A}\mathcal{W}\mathcal{I}(\mathcal{G}-{p}_{1})=0.2804.$$

As we remove the vertex ${p}_{2}$ from a graph, the nature of the remaining arcs also changed.

$${ds}_{\underline{\mathcal{G}}}({p}_{1},{p}_{3})=(\text{0.5,0.1}), {ds}_{\underline{\mathcal{G}}}({p}_{3},{p}_{1})=(\text{0.5,0.1}), {ds}_{\overline{\mathcal{G}}}({p}_{1},{p}_{3})=(\text{0.5,0.2}), {ds}_{\overline{\mathcal{G}}}({p}_{3},{p}_{1})=(\text{0.5,0.2}),$$

$$\mathcal{W}\mathcal{I}(\underline{\mathcal{G}}-{p}_{2})=0.1947, \mathcal{W}\mathcal{I}(\overline{\mathcal{G}}-{p}_{2})=0.0857, \mathcal{W}\mathcal{I}(\mathcal{G}-{p}_{2})=0.2804.$$

$$\mathcal{A}\mathcal{W}\mathcal{I}(\underline{\mathcal{G}}-{p}_{2})=0.1947, \mathcal{A}\mathcal{W}\mathcal{I}(\overline{\mathcal{G}}-{p}_{2})=0.0857, \mathcal{A}\mathcal{W}\mathcal{I}(\mathcal{G}-{p}_{2})=0.2804.$$

As we remove the vertex ${p}_{3}$ from a graph, the nature of the remaining arcs also changed.

$${ds}_{\underline{\mathcal{G}}}({p}_{1},{p}_{2})=(\text{0.0,0.0}), {ds}_{\underline{\mathcal{G}}}({p}_{2},{p}_{1})=(\text{0.6,0.2}), {ds}_{\overline{\mathcal{G}}}({p}_{1},{p}_{2})=(\text{0.0,0.0}), {ds}_{\overline{\mathcal{G}}}({p}_{2},{p}_{1})=(\text{0.5,0.2}),$$

$$\mathcal{W}\mathcal{I}(\underline{\mathcal{G}}-{p}_{3})=0.1947, \mathcal{W}\mathcal{I}(\overline{\mathcal{G}}-{p}_{3})=0.0857, \mathcal{W}\mathcal{I}(\mathcal{G}-{p}_{3})=0.2804.$$

$$\mathcal{A}\mathcal{W}\mathcal{I}(\underline{\mathcal{G}}-{p}_{3})=0.1947, \mathcal{A}\mathcal{W}\mathcal{I}(\overline{\mathcal{G}}-{p}_{3})=0.0857, \mathcal{A}\mathcal{W}\mathcal{I}(\mathcal{G}-{p}_{3})=0.2804.$$

### Application: transport network flow

The Wiener index is a useful tool for analyzing transport network flow, assessing its robustness and efficiency under different conditions. It quantifies the shortest path distances between nodes, allowing for the identification of the impact of road closures on the network’s connectivity and functionality. This approach helps identify critical routes that could disrupt the network and evaluate alternative pathways for operational continuity.

**Fig. 11:**
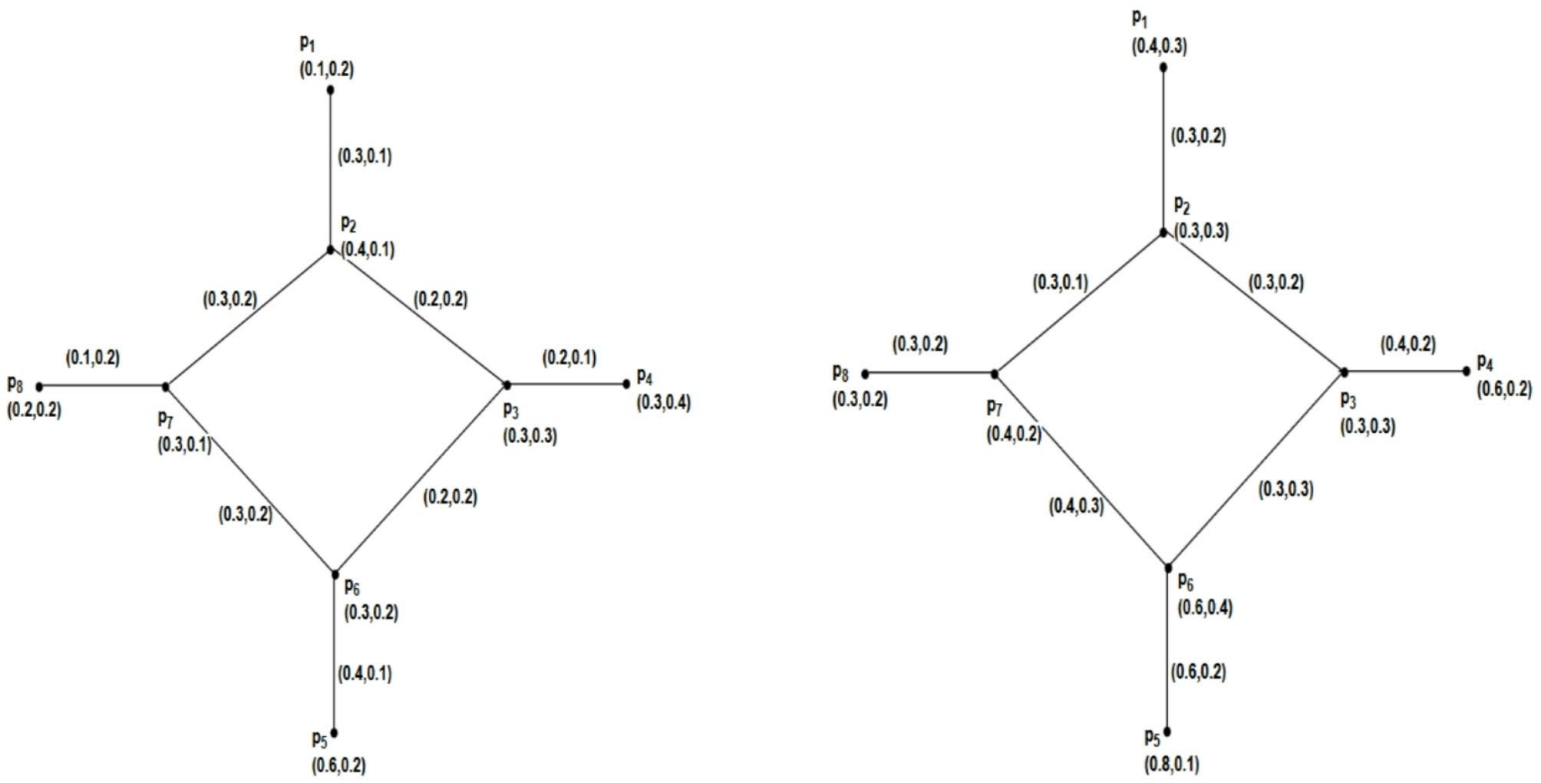
Intuitionistic fuzzy rough transport network flow.

Figure 11. illustrates a transport network flow, with vertices representing cities and edges representing roads connecting them. Each vertex represents the incoming and outgoing traffic flows associated with that city, reflecting its hub role. The edges represent the flow of traffic along the roads, capturing the intensity and direction of movement between cities. This detailed representation helps analyze traffic distribution, congestion points, traffic dynamics, and potential disruptions like road closures or rerouting strategies.

In this network, each arc is robust, and through straightforward calculation, we have complied the following table.

From Table 2, it is evident that certain cities namely ${p}_{1},{p}_{4},{p}_{5},and {p}_{8}$​ become isolated when specific arcs or roads are removed, such as $\left({p}_{1},{p}_{2}\right)$,$\left({p}_{3},{p}_{4}\right)$,$\left({p}_{5},{p}_{6}\right)$, and $\left({p}_{7},{p}_{8}\right)$. This isolation results in these cities being disconnected from their neighboring cities, specifically ${p}_{2}$, ${p}_{3},$ ${p}_{6},$ and ${p}_{7}$​. Consequently, the removal of these edges impacts the network by reducing its overall Wiener index, which measures the efficiency of connections across the network. The Wiener index has decreased, but the network still shows a significant difference in incoming and outgoing traffic paths. This indicates a natural asymmetry in the network’s traffic flow distribution, suggesting it is designed to accommodate more incoming traffic than outgoing ones, indicating a predominant incoming traffic flow.

**Table 2:** Wiener index for $\mathcal{G}$ and $\mathcal{G}-\left({p}_{1},{p}_{2}\right)$, and $\mathcal{C}\mathcal{I}(\mathcal{G})$.

Arcs	Types of arcs	Graphs	$\mathcal{W}\mathcal{I}(\mathcal{G})$	$\mathcal{C}\mathcal{I}(\mathcal{G})$
–	–	$\mathcal{G}$	8.1450	6.8590
$\left({p}_{1},{p}_{2}\right)$	Strong arc	$\mathcal{G}-\left({p}_{1},{p}_{2}\right)$	6.1160	5.6190
$\left({p}_{2},{p}_{3}\right)$	Strong arc	$\mathcal{G}-\left({p}_{2},{p}_{3}\right)$	8.3730	6.9580
$\left({p}_{3},{p}_{4}\right)$	Strong arc	$\mathcal{G}-\left({p}_{3},{p}_{4}\right)$	5.7120	4.3390
$\left({p}_{3},{p}_{6}\right)$	Strong arc	$\mathcal{G}-\left({p}_{3},{p}_{6}\right)$	8.9250	7.0101
$\left({p}_{5},{p}_{6}\right)$	Strong arc	$\mathcal{G}-\left({p}_{5},{p}_{6}\right)$	4.3550	3.1080
$\left({p}_{6},{p}_{7}\right)$	Strong arc	$\mathcal{G}-\left({p}_{6},{p}_{7}\right)$	9.1283	7.4688
$\left({p}_{7},{p}_{8}\right)$	Strong arc	$\mathcal{G}-\left({p}_{7},{p}_{8}\right)$	5.4800	3.3844
$\left({p}_{2},{p}_{7}\right)$	Strong arc	$\mathcal{G}-\left({p}_{2},{p}_{7}\right)$	9.2260	7.5447

Table 2 indicates that $\mathcal{W}\mathcal{I}(\mathcal{G}-\left({p}_{1},{p}_{2}\right))$ $> \mathcal{W}\mathcal{I}(\mathcal{G})$, implying that the removal of the arc $\left({p}_{1},{p}_{2}\right)$ increases the Wiener index of the network. This increase occurs because eliminating this arc results in some routes becoming longer than they were previously. For instance, the geodesic route from ${p}_{1}$ to ${p}_{3}$​, which originally followed the path ${p}_{1}{p}_{2}{p}_{3}$​ with a length of 2, now takes the longer path ${p}_{1}{p}_{2}{p}_{7}{p}_{6}{p}_{3}$​, increasing its length to 4. Along with the increased length, the total weight of this route has also risen, reflecting higher costs in terms of time and money for travellers journeying from city ${p}_{1}$ to ${p}_{3}$​. Similarly, the removal of the arc $\left({p}_{1},{p}_{2}\right)$ affects other routes, such as those from ${p}_{1}$ to ${p}_{4}$​ and ${p}_{2}$ to ${p}_{3}$​, making them longer and potentially more expensive. However, not all routes are impacted by this change. For example, the route from ${p}_{2}$ to ${p}_{5}$​ remains unaffected, as there are still two geodesics of equal length between these cities. This availability of alternative routes with unchanged geodesic distances ensures that travellers between ${p}_{2}$ and ${p}_{5}$ do not face significant inconvenience following the removal of $\left({p}_{2},{p}_{3}\right)$. Similar observations can be made for the removal of other arcs, such as $\left({p}_{3},{p}_{6}\right)$, $\left({p}_{6},{p}_{7}\right)$, and $\left({p}_{2},{p}_{7}\right)$. In each case, the analysis identifies how the deletion of specific arcs alters the connectivity and efficiency of the transport network, particularly focusing on changes in route lengths, costs, and the availability of alternative paths. This analysis assesses the impact of closing or removing specific routes on connectivity, travel efficiency, and resource allocation, providing valuable insights for efficient transport network management.

A comparison of the Wiener index and the connectivity index, as presented in Table 2, reveals that the values of these two indices do not align for both the original network $\mathcal{G}$ and the modified network $\mathcal{G}-\left({p}_{i},{p}_{j}\right)$, even though all the roads in the network are robust. This discrepancy arises because the network does not provide a direct road connection between every pair of cities. Cities ${p}_{2} and {p}_{6}$ are not directly connected by road, affecting the calculation of the Wiener index and connectivity index differently. The Wiener index accounts for shortest path distances, while the connectivity index measures the network’s robustness and redundancy. This lack of agreement highlights the network’s structural characteristics, highlighting that the absence of direct connections affects efficiency and connectivity differently. Understanding these differences is crucial for understanding the network’s topology and functionality. It is also noteworthy that both indices show lower values than before when strong roads are removed, indicating a similar behaviour. However, when strong roads are deleted, the values of the indices increase compared to their original values. Specifically, the Wiener index rises more significantly than before, while the connectivity index remains unchanged.

### Advantages

Our study offers several key advantages. First, the primary benefit of our work is the development of the $\mathcal{W}\mathcal{I}$ for IFRGs settings, which are classified into two categories: membership and non-membership approximations. This dual approach provides a richer representation of relationships compared to traditional fuzzy graphs. Second, IFGs enhance this by incorporating both membership and non-membership degrees, allowing for a more detailed and nuanced depiction of relationships. IFRGs take this a step further by integrating rough set theory, making them particularly effective for modeling uncertainty and imprecise relationships in complex systems. Lastly, our research demonstrates that IFRGs offer more information than IFGs, thereby providing a more robust framework for analyzing and processing uncertain or incomplete data.

## Conclusion

One of the mathematical tools for analyzing a graph’s structural characteristics is a topological index. Topological indices handle numerous graph theory issues. In crisp and fuzzy graph theory, there exist a number of topological indices; however, these models are not applicable to all graphical structures in all situations. The aim of this research is to apply the wiener index ($\mathcal{W}\mathcal{I}$) idea generally to IFRGs. Sometimes the features of two graphs cannot be distinguished by the analysis of the connection index in many IFRG issues. In order to get around this problem, we apply the wiener index notion. In this work, we introduced the idea of the wiener index, a unique idea for an IFRG that is based on the separation between its vertices. This study studies the link between an IFRG’s wiener index and connectivity index ($\mathcal{C}\mathcal{I}$) through a number of cases. Furthermore, we discovered a few other IFRG indices. We have established these indexes to lay the framework for a more thorough comprehension of the features and intricacies of IFRGs. The average wiener index ($\mathcal{A}\mathcal{W}\mathcal{I}$), which is determined by averaging the distances between the IFRG’s vertices, has been a topic of discussion. Following that, a crucial application of the wiener index to the transport network flow was given. In the future, we may create these indices with the intuitionistic fuzzy rough hypergraph taken into account as well, and we can see how that enhances the definitions of the indices that are already specified in the study. We also aim to introduce some other indices in IFRGs and investigate their applications.

## Data Availability

All data generated or analysed during this study are included in this article.
